# Metallothionein 2 regulates endothelial cell migration through transcriptional regulation of *vegfc* expression

**DOI:** 10.1007/s10456-015-9473-6

**Published:** 2015-07-22

**Authors:** Annika Schuermann, Christian S. M. Helker, Wiebke Herzog

**Affiliations:** University of Muenster, Muenster, Germany; Cells-in-Motion Cluster of Excellence (EXC 1003 – CiM), University of Muenster, Muenster, Germany; Max-Planck-Institute for Heart and Lung Research, Bad Nauheim, Germany; Max-Planck-Institute for Molecular Biomedicine, Muenster, Germany

**Keywords:** Vegfc, Angiogenesis, Endothelial cell migration, TALEN, Nonsense-mediated decay, Phenotype variability, Zebrafish

## Abstract

Analysis of developmental angiogenesis can help to identify regulatory networks, which also contribute to disease-related vascular growth. Vascular endothelial growth factors (Vegf) drive angiogenic processes such as sprouting, endothelial cell (EC) migration and proliferation. However, how Vegf expression is regulated during development is not well understood. By analyzing developmental zebrafish angiogenesis, we have identified Metallothionein 2 (Mt2) as a novel regulator of *vegfc* expression. While Metallothioneins (Mts) have been extensively analyzed for their capability of regulating homeostasis and metal detoxification, we demonstrate that Mt2 is required for EC migration, proliferation and angiogenic sprouting upstream of *vegfc* expression. We further demonstrate that another Mt family member cannot compensate Mt2 deficiency and therefore postulate that Mt2 regulates angiogenesis independent of its canonical Mt function. Our data not only reveal a non-canonical function of Mt2 in angiogenesis, but also propose Mt2 as a novel regulator of *vegfc* expression.

**Electronic supplementary material:**

The online version of this article (doi:10.1007/s10456-015-9473-6) contains supplementary material, which is available to authorized users.

## Introduction

Growth of blood vessels during development as well as in the adult organism is a tightly regulated process, which is controlled by endothelial cell (EC) behaviors such as cell migration, proliferation and differentiation. Misregulation of vascular growth not only contributes to ischemic conditions, but overgrowth also directly aggravates diseases such as growth and metastasis of cancers or age-related macular degeneration.

Vascular endothelial growth factors (Vegfs) and their Vegf receptors (VEGFR-1/Flt1, VEGFR-2/Kdrl-Kdr and VEGFR-3/Flt4) are the major regulators of vascular growth processes [1–3].

While VEGFA and VEGFR-2 (Kdrl in zebrafish) mainly regulate angiogenic processes such as sprouting and remodeling of vessels [4, 5], VEGFC and VEGFR-3 have mainly been recognized for their role in regulating development of the lymphatic endothelial system [6–8]. *Vegfr3*/*flt4*-deficient zebrafish completely lack lymphatic vessels but show no major defects in blood vessel growth [9]. VEGFR3-deficient mice die of defective vascular development before the lymphatic system becomes established [10]. *Vegfc* mutant mice as well as zebrafish lack a lymphatic system [11, 12]. Angiogenesis defects observed in *vegfc* zebrafish mutants include failure in EC migration [during formation of the primordial hindbrain channels (PHBCs)] [11] as well as reduced EC proliferation [in the common cardinal veins (CCVs)] [13].

However, VEGFA as well as VEGFC expression are both upregulated in various tumors and their misregulation is involved in other diseases; therefore, understanding the mechanisms regulating their expression are of clinical relevance [14, 15].

Within cultured fibroblast or cancer cells*, VEGFC* mRNA expression was shown to be upregulated by cytokines (Interleukin-1α or interleukin-1β, or Tumor necrosis factor-α) [16] and growth factors (Platelet derived growth factor, Epidermal growth factor and Transforming growth factor-β) [17], but not by Hypoxia-inducible factor-1α (HIF1α) [18].

The optical clarity of the externally developing zebrafish embryos is one of the many advantages for using this model for the analysis of vascular development. The growing vasculature can easily be visualized in vivo by endothelial-specific transgenic fluorophore expression [19].

The vascular anatomy of zebrafish embryos has a high structural homology to other vertebrates [20, 21]. Similarly, most signaling pathways regulating vascular development are conserved between zebrafish and mammals [22, 23]. A functional circulatory system including a primitive heart is already established in the zebrafish embryo by 24 h post-fertilization (hpf).

Additionally, recent advances in genome editing using Transcription activator-like effector nucleases (TALENs) or Cas9 nucleases [24, 25] enabled gene-specific targeting in zebrafish.

We performed gene expression analyses to identify novel regulators of angiogenesis in zebrafish embryos and thereby identified *metallothionein 2* (*mt2*) as a candidate.

MTs are low-molecular-weight and cysteine-rich proteins, which are conserved throughout the animal kingdom. There are four classes of mammalian *Mt* genes, *Mt1*–*4* [26, 27], and two *mt* genes in zebrafish, *mt2* and *metallothionein*-*B*-*like* (*mtbl*) [28, 29].

The main function of MTs is the regulation of homeostasis, such as the protection against oxidative stress or metals. Both heavy and trace metals such as zinc, copper or iron can be chelated via sulfur-based clusters [30, 31].

However, MTs also display non-canonical functions in angiogenesis and pathological conditions. *Mt1* and *Mt2* are very similar and the best characterized genes of the MT family, which can act as tumor suppressors [32] and have cardio- and neuroprotective functions [33–35]. Mice deficient for both *Mt1* and *Mt2* are viable and show beside their greater sensitivity to metals no major developmental defects [36, 37]. When challenged by femoral artery ligation or cortical freeze injury, these *Mt1/2* knockout mice show impaired angiogenesis and wound healing [38–40]. MT3 is important for cell growth [41], and its expression is downregulated in a carcinoma cell line [42]. MT3 also has a critical role in the recovery of the brain, since *Mt3*-deficient mice show increased oxidative stress and apoptosis upon cortical freeze lesion [43]. The non-inducible *Mt4* is expressed in epithelial tissues and has only been shown to detoxify of metals [30, 44].

However, how MTs exert their non-canonical functions, such as the regulation of angiogenic processes, is not understood.

Here, we used zebrafish as a model to analyze the role of Mt in angiogenesis. We generated Mt2-deficient zebrafish embryos by performing antisense morpholino-oligonucleotide (MO)-mediated gene knockdown as well as by using TALEN to generate zebrafish *mt2* mutants. Using in vivo time-lapse analysis, we show that *mt2* deficiency leads to striking angiogenesis defects, especially to defective formation of the PHBCs. Furthermore, we demonstrate that Mt2 acts upstream of *vegfc* expression in regulating EC migration and proliferation. This regulation of angiogenesis represents a non-canonical function of Mt2, since another Metallothionein family member (Mtbl) cannot regulate *vegfc* expression.

## Materials and methods

### Zebrafish maintenance and strains

Zebrafish embryos were maintained at 28.5 °C under standard husbandry conditions [45]. Zebrafish lines used were *Tg*(*kdrl:EGFP*)^*s843*^ [46], *Tg*(*fli1a:EGFP*)^*y1*^ [47] and *Tg*(*fli1a:nEGFP*)^*y7*^ [48]. The *vegfc*^*hu6410*^ allele encodes a stop codon at amino acid position 107 (L107X) [49].

### Generation of the *mt2* mutant transgenic zebrafish line using transcription activator-like effector nucleases (TALENs)

TALENs were assembled using the Golden Gate method [50]. For targeting the *mt2* locus, a 5′ RVD (NH–NG–NH–NH–NI–NG–NI–HD–NG–HD–NG–HD–NG–NH (DNA sequence GTGGATACTCTCTGG)) and a 3′ RVD (NI–HD–NG–HD–NG–NG–NH–NH–HD–NI–HD–NI–NG–NG (DNA sequence ACTCTTGGCACATTC)) were generated with a spacer of 16 bp (AAAAATGGACCCCTGC) to target exon 1. An AvaII (New England BioLabs) restriction site within the spacer region was used for genotyping of putative founders. mRNA was generated using the T3 mMessage mMachine Kit (Ambion) and injected using 100 pg of the TALEN mix.

### mRNA and morpholino (MO) injections

MOs blocking either translation (MO) or RNA splicing (spbMO) were obtained from Gene Tools and are as follows:

*mt2* MO: 5′-GGTCCATTTTTCCAGAGAGTATCCT (5 ng) and *mt2* spbMO: 5′-AGCTGAAACACTTACTCTTGGCACA (7–10 ng), targeting *mt2* (BC152694.1); *mtbl* MO: 5′-CTGGTCCATCTTTACACCGTAGGTC and *mtbl* spbMO: 5′-AGTTAATCGGCTCACTTTTCTTGTC (both 13 ng) targeting *mtbl* (NM_001201469.1), *upf1* spbMO: 5′-TTTTGGGAGTTTATACCTGGTTGTC (0.1 ng) [51] and *smg1* spbMO: 5′-AACCATTGGTTTGTTACCTGGTGCA (12.5 ng) [51] and standard control MO: 5′-CCTCTTACCTCAGTTACAATTTATA.

For overexpression experiments, the *mt2* sequence was amplified from 24 hpf cDNA and cloned into the pCS2+ vector for in vitro RNA synthesis using the following primers: *mt2fwd* 5′-AGACGAATTCGCTCCACCATGGACCCCTGCGAATGTGC and *mt2rev* 5′-AGACCTCGAGTCATTGACAGCAGCTGGAGC.

Similarly, *mtbl* was cloned into the pCS2+ vector using *mtblfwd* 5′-AGACGAATTCGCTCCACCATGGACCAGTGTAACTGCTC and *mtblrev* 5′- AGACCTCGAGTCATTTGCAGCAGTGTGTGG.

The mRNA was synthesized using SP6 mMessage mMachine Kit (Ambion). For all experiments, the injection was done into the yolk of 1-cell-stage zebrafish embryos, and 0.05 % phenol red (Sigma) was added to the injection solution.

Injection amounts per embryo were as follows: 500 pg *mt2* mRNA, 100 pg *mtbl* mRNA, 100 pg to 500 pg *H2B*-*cherry* mRNA [52], 200 pg *vegfc* mRNA [53] and 200 pg *sFLT4* mRNA [54].

### RNA and DNA isolation, qPCR analysis and genotyping

RNA from WT, mutants and MO-injected embryos was isolated with Trizol reagent, and cDNA was generated by SuperScript II reverse transcriptase (Invitrogen).

The cDNA was analyzed with real-time quantitative PCR (qPCR) using Power SYBR Green (Applied Biosystems) and the following primers: *b*-*actinfwd* 5′-CTGGACTTCGAGCAGGAGAT and *b*-*actinrev* 5′-GCAAGATTCCATACCCAGGA (156 bp amplicon); *vegfcfwd* 5′-GCAGGAACATCAGCACTTCA and *vegfcrev* 5′-GTGTGGTTGGCGAAGCTTAT (103 bp amplicon); *fli1afwd* 5′-CTCAGGGAAAGTAGCTCATCG and *fli1arev* 5′-CTTTTCCGCTGTGCATGTT (139 bp amplicon); *myod1fwd* 5′-TCTGATGGCATGATGGATTT and *myod1rev* 5′-TTATTATTCCGTGCGTCAGC (110 bp amplicon). For qPCR at least two different cDNA samples were generated and analyzed. Experiments were performed at least three times.

The knockdown efficiency of the *mt2* splice MO was validated with reverse transcription PCR (RT-PCR) and primers *mt2fwd* 5′-ATGGACCCCTGCGAATGTGC and *mt2rev* 5′-TCTTCTTGCAGGTAGTACACTG (spliced amplicon 91 bp, non-spliced amplicon 185 bp). The functionality of the *mtbl* splice MO was analyzed with *mtblfwd* 5′-GACCAGTGTGACTGCTCCAA and *mtblrev* 5′-TGCAGGATTTCTCCTTGTCC (spliced amplicon 169 bp, non-spliced amplicon 327 bp).

DNA was extracted using lysis buffer (10 mM Tris–HCl, 50 mM KCl, 0.3 % Tween 20, 0.3 % Nonidet P-40, pH 8.3) with 0.5 μg/μl proteinase K (Roche) overnight at 55 °C, followed by 10 min denaturation at 95 °C.

The genotype of the *mt2*^*mu290*^, the *mt2*^*mu292*^ and the *mt2*^*mu289*^ mutants was analyzed with primers *mt2fwd* 5′-TCTTCTTGCAGGTAGTACACTG and *mt2rev* 5′-TAAAAGCAGAGCACAAACACG and the restriction enzyme AvaII.

The genotype of the *vegfc*^*hu6410*^ zebrafish mutants was analyzed in a multiplex PCR with WT and mutant zebrafish-specific inner and outer primers. As inner primers *vegfcfwd* 5′-CTTTCATCAATCTTGAACTTTT (WT specific) and *vegfcrev* 5′-TAAATTAATAGTCACTCACTTTACT (mutant specific with one mismatch) were used and as outer primers *vegfcfwd* 5′-GATGAACTCATGAGGATAGTTT and *vegfcrev* 5′-AAACTCTTTCCCCACATCTA.

### Whole-mount in situ hybridization

Whole-mount in situ hybridization was performed as described [55]. The *mt2* probe was amplified from 24 hpf zebrafish embryo cDNA with the T7 promoter site and the following primers: *mt2fwd* 5′-GGAACTTTCAAGCTCTTTGTGG and *mt2rev* 5′-gTAATACgACTCACTATAggGACAAAGGACATGGCAGAAAA. The *vegfc* probe is already described [53].

### Confocal microscopy and in vivo time-lapse analysis

Zebrafish embryos were analyzed with confocal microscopy as previously described, using 1 % agarose embryo moulds [56]. The fluorescent images were acquired using the Sp5 DM 6000 upright confocal microscope (Leica) or the inverse LSM 780 confocal microscope (Zeiss).

### BrdU incorporation and immunohistochemistry

Proliferation analysis was performed as described [57] with following modifications: Embryos were grown to 24 hpf and then incubated in 10 mM 5-bromo-2′-deoxyuridine (BrdU) for 30 min on ice. After 8 h of further incubation and BrdU incorporation, embryos were fixed in 4 % paraformaldehyde (PFA) at 32 hpf. After incubation in 2 M HCl for 1 h, permeabilization (phosphate-buffered saline (PBS) with 0.3 % Triton X−100 (Sigma) and 0.1 % Tween 20 (Sigma)) and blocking (PBS with 0.3 % Triton X−100 and 4 % BSA (Roth)), the following antibodies were used for immunostaining: mouse anti-BrdU (1:100, Roche), Alexa 546 anti-mouse (1:500, Invitrogen) and Alexa 488 anti-GFP (1:500, Invitrogen, for ECs of *Tg*(*kdrl:EGFP*)^*s843*^). After each antibody incubation, extensive washing was performed (PBS with 0.3 % Triton X−100).

### Phenotypic analysis, quantifications, statistics and softwares

For evaluation of the rescue experiments, different clutches of at least three different experiments were scored for the existence of the PHBCs. If the PHBCs were not present at all or developed to <50 %, they were considered as missing; if the PHBCs were developed to more than 50 % or fully connected, they were considered as existent. For rescue experiments of *vegfc*^*hu6410*^ zebrafish mutants, only embryos with a strong PHBC phenotype or with fully developed PHBCs were taken for analysis for both Ctr and *mt2* mRNA-injected zebrafish, and each embryo was subjected to subsequent genotyping. Cell numbers of fixed *Tg*(*kdrl:EGFP*)^*s843*^ or *Tg*(*fli1a:nEGFP*)^*y7*^ zebrafish embryos were evaluated with help of the Spots function of Imaris. Cells in the PHBC, the anterior cluster and the posterior clusters were counted at 32 hpf in confocal stacks. Similarly, ECs of the ACV, PCV and CCV were counted at 32 hpf, while ECs of the Ses were counted at 48 hpf.

For analysis of the amount of proliferating cells in the CCVs, BrdU-positive cells were calculated relative to the total number of ECs in the CCVs. For quantifying the Ses, the region between somites 9 and 14 has been analyzed. The *P* values for the experiments were calculated with a two-tailed Student’s *t*-test. The rescue experiments for the PHBC phenotypes were evaluated for significance with the Chi-Square test using Microsoft excel. SDS 2.3 and RQ Manager (Applied Biosystems) were taken for analysis of the real-time data. Primers were designed using Primer3 (http://bioinfo.ut.ee/primer3-0.4.0/primer3/input.htm).

Where possible, the analysis was performed blind to experimental conditions.

## Results

### Mt2 regulates EC behavior during angiogenesis

To identify regulators of EC migration, we screened for functional involvement of candidate genes using morpholino antisense oligonucelotides (MO) to knockdown protein expression in zebrafish embryos and analyzed their vascular development using endothelial-specific GFP expression (*Tg*(*kdrl:GFP*)^*s843*^).

**Fig. 1:**
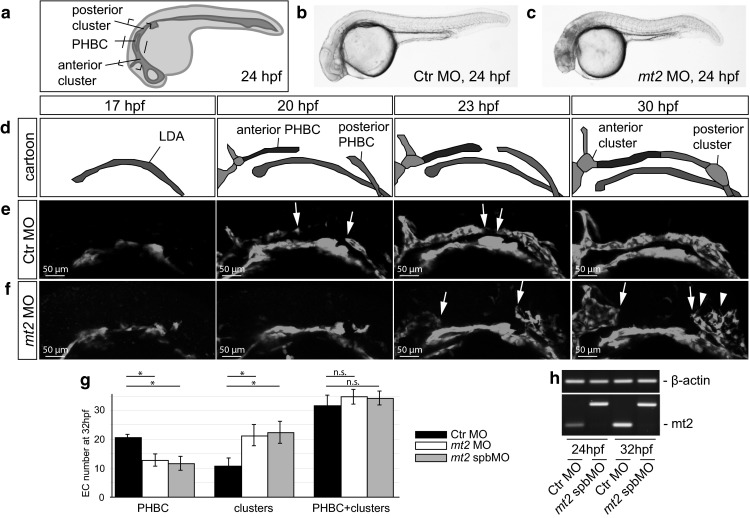
*mt2* morphants fail to form the PHBCs. **a** Schematic illustration of the vasculature of a 24 hpf old zebrafish embryo with the location of the PHBC and the adjacent PHBC forming clusters. **b, c **Brightfield images of Ctr (**b**) and *mt2* (**c**) MO-injected zebrafish embryos at 24 hpf showed no morphological defects, apart from a mild necrosis in the head. **d** Schematic illustration of the development of the PHBC (*dark blue*) between 17 and 30 hpf. **e**, **f** Confocal micrographs from time-lapse movies showing the development of the PHBC in zebrafish embryos between 18 and 30 hpf. The vasculature was visualized by transgenic GFP expression using *Tg*(*kdrl:EGFP*)^*s843*^ embryos. In embryos injected with Ctr MO, ECs migrate from the anterior and the posterior cluster and connect to form the PHBC before 24 hpf (at around 23 hpf; **e**). In embryos injected with *mt2* MO ECs fail to migrate and therefore do not form the PHBCs (**f**). *White arrows* indicate the anterior and posterior migration front of the PHBC. *White arrowheads* indicate filopodia in *mt2* morphants. **g** Quantification of EC numbers in Ctr MO-injected (*black bars*) or *mt2* MO-injected (*white bars*) and *mt2* spbMO-injected (*gray bars*) embryos counted from vascular-specific nuclear GFP expression (*Tg*(*fli1a:nEGFP*)^*y7*^). While the total EC numbers were not affected, *mt2* MO-injected embryos showed fewer ECs in the PHBC and more ECs in the clusters. *n* = 20, *P < 0.05; n.s., not significant; *error bars* indicate standard error of the mean (SEM). **h** Analysis of *mt2* splicing efficiency in embryos injected with Ctr MO or *mt2* spbMO. RT-PCR analysis showed a 185 bp amplicon in embryos injected with *mt2* spbMO, while functional splicing led to a 91 bp amplicon in Ctr MO-injected embryos. (Color figure online)

We identified Mt2 as a potential regulator of EC migration. For a detailed analysis, we injected MOs either inhibiting mRNA translation (using MO covering the ATG) or blocking mRNA splicing (spbMO). Embryos injected with *mt2* MO or *mt2* spbMO, showed brain necrosis but no other major morphological defects (Fig. 1). Of the affected vessels, the PHBCs are the first to develop. They grow by angiogenic sprouting out of an anterior cluster and a posterior cluster of ECs, which start at 18 hpf to migrate toward each other and connect around 22–23 hpf to form a functional vessel (Fig. 1d, e; movie 1). At 24–25 hpf, circulation starts and blood flow can be observed going through the PHBCs. However, we observed not only defective growth of the PHBCs, but also of the CCVs and the Ses at different time points of development (Fig. 1, Fig. S1).

We used in vivo time-lapse imaging to further analyze PHBC formation in control MO (Ctr)- or *mt2* MO-injected *Tg*(*kdrl:EGFP*)^*s843*^ embryos. The Ctr and *mt2* MO-injected (morphant) embryos were indistinguishable from each other until 18 hpf (Fig. 1e, f), with both displaying normal development of the lateral dorsal aorta (LDA). In Ctr morphants, the ECs migrated, connected and thereby formed the PHBCs (Fig. 1e, movie 1), whereas *mt2* morphant ECs failed to migrate out of the clusters and did not connect to form the PHBC (Fig. 1f, movie 2). However, the ECs were motile and formed filopodia, but the directed migration required for the connection of the PHBCs was perturbed (movie 2). Some *mt2-*deficient embryos extended sprouts from the anterior and posterior cluster to develop the PHBCs, but no proper connection was established. To determine, whether this defect is caused by defective migration or reduced EC numbers, we counted the number of ECs in the PHBCs as well as in the anterior and posterior cluster at 32 hpf, long after PHBC formation should have been completed. While the total EC number in PHBCs and clusters was similar, Ctr morphants had an average of 21 cells in the PHBC and 12 cells in the clusters, whereas *mt2* morphants had an average of 13 cells in the PHBC and 22 cells in the cluster (Fig. 1g). Therefore, our results indicate that Mt2 regulates EC migration during PHBC angiogenesis.

Additionally, we analyzed CCV formation in Ctr and *mt2* morphants in more detail (Fig. S1). The CCVs grow at a 90 °C angle out of the trunk ACV and posterior PCV, by a combination of collective EC migration and proliferation [13]. At 32 hpf the total cell number in ACV, PCV and CCV was reduced in *mt2* morphants compared with Ctr morphants (Fig. S1j). However, the percentage of cells in the CCV was significantly reduced from 35 % CCV cells in Ctr embryos to 26 % in mt2-deficient embryos (Fig. S1i). To test whether Mt2 regulates EC migration or proliferation in the CCV, we performed proliferation analysis by BrdU incorporation in *mt2* morphants. Proliferation was strongly decreased in *mt2* morphants (Fig. S2).

In sum, our results indicate that Mt2 regulates angiogenesis, by regulating EC migration in the PHBCs and EC proliferation during CCV formation.

### *mt2* zebrafish mutants phenocopy the *mt2* morphants

**Fig. 2:**
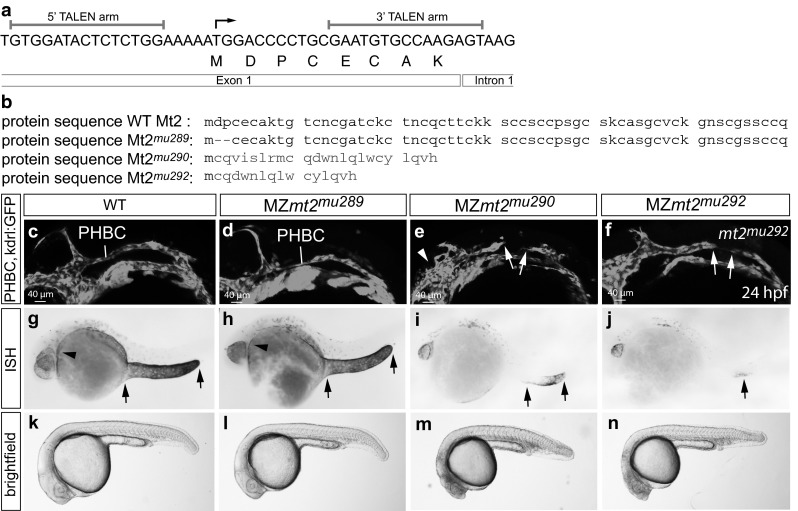
TALEN-generated *mt2* zebrafish mutants fail to form the PHBC and have different levels of NMD of *mt2* transcripts. **a** 5′ and 3′ TALEN arms were designed to target exon 1 of the *mt2* gene to induce mutations in the genome. **b** TALEN injection resulted in various genomic mutations. Illustrated is the comparison of the amino acid sequence in WT and different Mt2 mutant alleles, *red color* indicates mutated amino acids. In *mt2* ^*mu289*^ mutants a 6 bp deletion resulted in deletion of two amino acids, while in *mt2* ^*mu290*^ and *mt2* ^*mu292*^ frameshift mutations resulted in complete changes of the amino acid sequence. **c**–**f** Confocal images of the PHBCs at 24 hpf. WT (**c**) and MZ*mt2* ^*mu289*^ mutant zebrafish embryos (**d**) form a PHBC, while MZ*mt2* ^*mu290*^ and *MZmt2* ^*mu292*^ mutant embryos fail to connect the PHBCs. The vasculature was visualized by transgenic GFP expression from *Tg*(*fli1a:EGFP*)^*y1*^ for MZ*mt2* ^*mu289*^ and MZ*mt2* ^*mu290*^ mutant embryos and from *Tg*(*kdrl:EGFP*)^*s843*^ for MZ*mt2* ^*mu292*^ mutant embryos. *White arrows* indicate the anterior and posterior migration front of the PHBC. *mt2* expression was analyzed by in situ hybridization in 24 hpf-old embryos. While WT siblings (**g**) and MZ*mt2* ^*mu289*^ mutants (**h**) had similar *mt2* expression levels, nonsense-mediated decay led to degradation of *mt2* mRNA transcript in MZ*mt2* ^*mu290*^ (**i**) and MZ*mt2* ^*mu292*^ (**j**) mutant embryos. *Black arrows* indicate *mt2* expression in cells of the yolk extension, *black arrowheads* label the region of the PHBCs. **k**–**n** Brightfield images of WT, MZ*mt2* ^*mu289*^, MZ*mt2* ^*mu290*^ and MZ*mt2* ^*mu292*^ mutant embryos at 24 hpf. WT siblings and MZ*mt2* ^*mu289*^ displayed no morphological defects. MZ*mt2* ^*mu290*^ and MZ*mt2* ^*mu292*^ mutant embryos are smaller in size and display necrosis in the head

Despite performing extensive control experiments, MOs have been shown to exhibit off-target effects [58–60]. To verify the phenotype of the *mt2* morphants, we used TALENs [50] to induce double-strand breaks in the *mt2* gene. As expected, errors made by the repair machinery of the cell then led to mutations in the double-strand break area [50]. Since the *mt2* sequence is very short, we targeted exon 1, which consists of 25 base pairs (bp) only (Fig. 2a). We identified several different alleles of *mt2* mutations and further analyzed three of them.

In the *mt2*^*mu289*^ mutant allele only 6 bp were deleted, which resulted in deletion of the second and third amino acid of the Mt2 protein (Fig. 2b, S3). The *mt2*^*mu290*^ sequence has two-point mutations and an insertion of 8 bp, which led to a frame shift and an early stop codon. The *mt2*^*mu292*^ sequence has a deletion of 15 bp, which lead to a frame shift and an early stop codon (Fig. 2b, S3). Since the mutations of *mt2*^*mu290*^ and *mt2*^*mu292*^ are located next to the start codon and there is no downstream start codon in frame, the original Mt2 protein sequence should be completely lost, supposedly resulting in null mutants.

By mating F1 heterozygous carriers of each *mt2* allele, we obtained homozygous F2 embryos. To our surprise, we only detected very weak phenotypes (data not shown). Since *mt2* is maternally provided [61], we hypothesized that the maternal mRNA is sufficient to rescue *mt2* deficiency during the early developmental stages analyzed. Therefore, we raised homozygous F2 embryos to adulthood. When mating homozygous *mt2* mutant fish to obtain maternal and zygotic mutant (MZ) F3 offspring, we observed strong morphological and angiogenesis phenotypes (Fig. 2), phenocopying the *mt2* morphants. Both MZ*mt2*^*mu290*^ and MZ*mt2*^*mu292*^ mutants failed to connect the PHBCs, had reduced cell numbers in the CCVs and defective Ses formation. (Fig. 2e, f; Fig. S4). Additionally, in a subset of *mt2* mutant embryos the morphology of the PHBC clusters was affected, with the ECs forming ectopic sprouts (arrowhead, Fig. 2e). The MZ*mt2*^*mu289*^ zebrafish mutants, which lack only two amino acids, displayed only a very weak phenotype. The PHBCs (Fig. 2d) and the Ses (Fig. S4g) developed normally in those mutants, while a mild phenotype could be observed in the CCVs (Fig. S4b).

The penetrance and severity of the phenotype for both null mutants were variable within the clutch and between clutches. MZ*mt2*^*mu290*^ zebrafish mutants showed severe phenotypes at higher rates than MZ*mt2*^*mu292*^ zebrafish mutants (compare Table 1), although both should not retain any amino acid sequence of Mt2. In order to investigate whether the mutations were causing strong alleles, we examined the level of gene transcription. One mechanism potentially interfering with mRNA transcript stability in mutants is nonsense-mediated decay (NMD), whereas MO-mediated blocking of translation would rather stabilize the transcript.

**Table 1:** *mt2* zebrafish morphants, MZ*mt2* zebrafish mutants and *vegfc* ^*hu6410*^ zebrafish mutants display many common phenotypes

	PHBCs	Clusters	Ses	CCVs
WT	0	2.26	2.53	4.75
*mt*2 MO	79.07	92.78	94.29	84.76
*mt* spbMO	75.00	85.71	90.24	77.78
MZ*mt2* ^*mu289*^	0	3.64	6.50	18.20
MZ*mt2* ^*mu290*^	34.22	84.42	90.37	41.63
MZ*mt2* ^*mu292*^	8.02	35.73	68.75	26.78
*vegfc* ^*hu6410*^	59.52	7.50	6.34	65.00

Overview of the frequencies of the different phenotypes observed upon *mt2* deficiency compared to *vegfc* deficiency and WT zebrafish embryos. The following classification of phenotypes was scored as affected: PHBCs: The PHBCs were developed to less than 50 % in length; cluster: severely thickened anterior cluster or additional ectopic sprouts or holes; CCVs: reduction by more than 15 % of EC numbers; Ses (scored between somites 9 and 14): Se sprouts were either significantly shortened by more than 15 % or Se numbers were reduced to less than 85 %. The PHBC, cluster and Se phenotypes were analyzed at 24 hpf; the CCVs were analyzed at 32 hpf (WT *n* = 138, *vegfc* ^*hu6410*^ *n* = 93, *mt2* MO *n* = 168, *mt2* spbMO *n* = 157, *mt2* ^*mu292*^ *n* = 256, *mt2* ^*mu290*^ *n* = 128, *mt2* ^*mu289*^ *n* = 123)

We therefore subjected 24 hpf-old MZ*mt2* mutant embryos to in situ hybridization to analyze the presence of the *mt2* transcript. While WT and MZ*mt2*^*mu289*^ mutant embryos expressed *mt2* as published [61], almost no expression could be observed in MZ*mt2*^*mu290*^ and MZ*mt2*^*mu292*^ mutants (Fig. 2g–j). Interestingly, the efficiency of NMD was not the same for both null mutants. While the great majority of MZ*mt2*^*mu292*^ embryos completely lacked *mt2* expression (Fig. 2j), some MZ*mt2*^*mu290*^ mutant embryos retained *mt2* message partially (Fig. 2i), which correlated with the different frequencies of angiogenesis defects (Table 1). We hypothesized that the more efficient the NMD was for the *mt2* zebrafish mutant, the more compensation mechanisms might take place to attenuate the phenotype. To analyze, whether mRNA stability could indeed influence the phenotypic severity, we partially ablated two subunits of the NMD mediating complex (*smg1* and* upf1*) by injecting *smg1/upf1* MOs in WT and in MZ*mt2*^*mu290*^ mutant embryos. We could indeed observe an increase in the number of affected embryos, when message degradation by NMD was reduced (Fig. S5). This indicates that indeed the correlation of the stronger phenotype with the reduced mRNA degradation is functionally relevant. The sum of this data implies that different levels of mRNA degradation can lead to differences in the phenotypes of generated zebrafish mutants and morphants, potentially by regulating unknown compensatory mechanisms.

### Mt2 acts upstream of Vegfc in regulating angiogenesis

Mt2 deficiency resulted in angiogenic defects during PHBC and CCV formation. Both of these processes have been described to be regulated by Vegfc during zebrafish embryonic development. Vegfc mutants or morphants fail to connect the PHBCs and have reduced proliferation in their CCVs [11, 13].

**Fig. 3:**
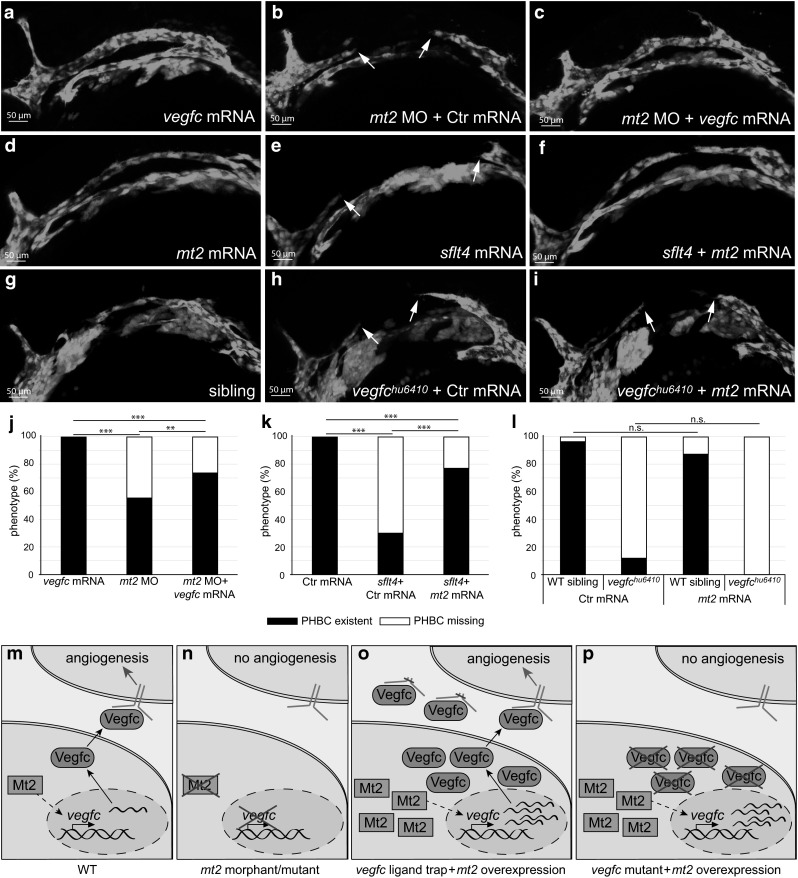
Mt2 acts upstream of Vegfc in PHBC formation. **a**–**c**, **j** Injection of *vegfc* mRNA rescued *mt2* deficiency in *mt2* morphants. Overexpression of *vegfc* mRNA does not disturb PHBC formation (**a**). Upon *mt2* MO injection (**b**), 44 % of the embryos lack the PHBC, while upon co-injection with *vegfc* mRNA (**c**) PHBC formation becomes rescued in half of the affected embryos (quantification of different experiments shown in **j**). **d**–**f**, **k** *mt2* overexpression rescued PHBC formation defects induced by overexpression of a Vegfc ligand trap (*sflt4* overexpression). Injection of *mt2* mRNA resulted in normal PHBC development (**d**). Depletion of Vegfc through injection of *sflt4* mRNA led to a failure in PHBC formation in 68 % of the embryos (**e**). Co-injection of both *mt2* and *sflt4* mRNA rescued PHBC formation and left only 23 % of embryos showing no PHBC (quantifications of different experiments shown in **k**). **g**–**i**, **l** Overexpression of *mt2* mRNA in embryos with a genetic null mutation in the *vegfc* gene (*vegfc* ^*hu6410*^) could not rescue the PHBC phenotype. Embryos were scored for their phenotype and subsequently genotyped for the *vegfc* mutation (quantifications of different experiments shown in **l**). The analysis was performed using *Tg*(*kdrl:EGFP*)^*s843*^ (**a**–**f**) and *Tg*(*fli1a:EGFP*)^*y1*^ (**g**–**i**) embryos. **j**–**k** Quantifications of the phenotypes observed after injection of indicated reagents: *Black bars* label percent of embryos with the PHBC formed, *white bars* label percent of embryos lacking the PHBC. Statistical significance was calculated with the Chi-square test, *n* = 228 (**j**), *n* = 277 (**k**), *n* = 124 (**l**), **P < 0.01; ***P < 0.001; n.s., not significant. **m**–**p** Schematics representing the proposed mechanisms of angiogenesis regulation in the experiments shown in **a**–**l**

We therefore carried out different rescue experiments to analyze whether there is an interaction of Mt2 and Vegfc signaling. We ubiquitously overexpressed Vegfc in WT or *mt2* morphant embryos by injection of *vegfc* mRNA into 1-cell-stage embryos. Overexpression of *vegfc* in WT embryos did not alter EC migration to form the PHBCs (Fig. 3a), but significantly reduced the number of embryos with PHBC connection defects from 44 % affected *mt2* morphants to 25 % affected *vegfc*-injected *mt2* morphants (Fig. 3c, j, n). Furthermore, we overexpressed the Vegfc ligand trap *sflt4* [54], which is a soluble form of the Vegfr3, that titrates away Vegfc and therefore results in the same phenotypes as the genetic *vegfc* mutation (Fig. 3e, k). By combining *sflt4* with high amounts of *mt2* mRNA injection, we could compensate the PHBC formation failure (Fig. 3f, k, o). Injection of *vegfc* mRNA rescued the *sflt4* mRNA injection to a similar extent (data not shown), indicating that Mt2 overexpression could indeed compensate for Vegfc ligand depletion. Interestingly, when we repeated the same experiment of rescuing Vegfc deficiency by *mt2* overexpression in *vegfc*^*hu6410*^ mutant embryos, Mt2 failed to rescue (Fig. 3i, l, p), suggesting that *vegfc* is the only relevant target of *mt2*. We confirmed the upregulation of *vegfc* transcripts after *mt2* mRNA injection in *vegfc*^*hu6410*^ mutant embryos with qPCR (Fig. S6). Taken together our data showed that Mt2 deficiency can be overcome by Vegfc addition and that Mt2 overexpression can outcompete Vegfc protein depletion, but not Vegfc mutation. These results are consistent with a mechanism, in which Mt2 regulates *vegfc* RNA expression (Fig. 3m).

### Mt2 regulates transcript levels of *vegfc*

**Fig. 4:**
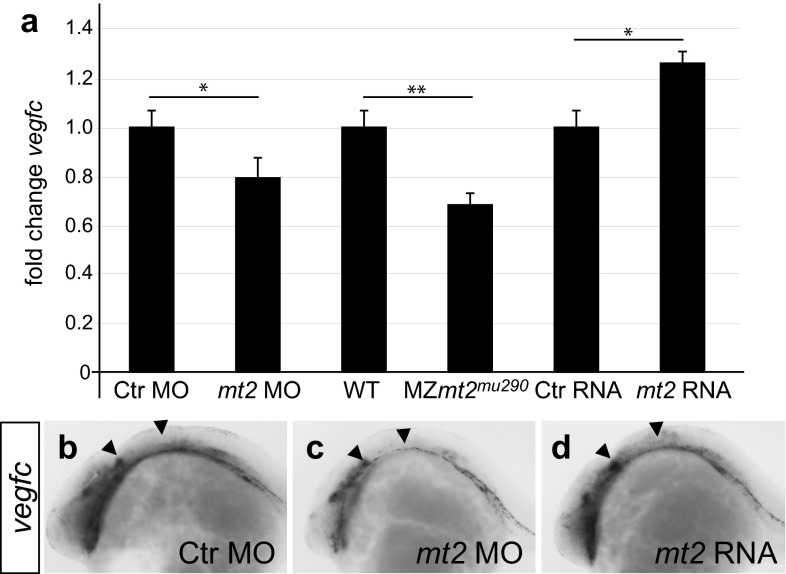
Mt2 regulates transcript levels of *vegfc*. **a** qPCR analysis of *vegfc* transcript levels (**a**) in *mt2* morphants, MZ*mt2* ^*mu290*^ mutants and *mt2* mRNA-injected embryos compared to Ctr embryos. Both morphants and mutants showed a significant decrease in *vegfc* transcript levels, while overexpression of *mt2* led to an increase in *vegfc* transcripts. *n* = 3, *P < 0.05; **P < 0.01; *error bars* represent SEM; **b**–**d** in situ hybridization for *vegfc* in hpf embryos injected with *mt2* MO or with *mt2* mRNA showed a reduction in *vegfc* expression upon knockdown of *mt2* (**c**) and an increase after *mt2* mRNA injection (**d**)

Given the results above, we used qPCR to analyze *vegfc* transcript levels in *mt2* morphant, MZ*mt2*^*mu290*^ mutant and *mt2*-overexpressing embryos. qPCR analysis revealed a 20 % decrease in *vegfc* RNA in *mt2* morphants and a 31 % decrease in MZ*mt2*^*mu290*^ mutant embryos (Fig. 4a). *mt2* overexpression on the other hand led to a 27 % increase in *vegfc* RNA levels in zebrafish embryos (Fig. 4a). To test whether *vegfc* transcripts are specifically affected, we analyzed further genes in *mt2-*deficient and *mt2*-overexpressing embryos. We chose *fli1a* as an EC-specific gene and *myod1*, a muscle-specific marker to represent other tissues [62]. We observed no significant changes in either *fli1a* or *myod1* transcript levels, irrespective of the *mt2* expression level. In contrast the significant changes in *vegfc* transcripts correlated with the changes in *mt2* expression as *vegfc* levels were decreased in *mt2*-deficient and increased in *mt2*-overexpressing cells (Fig S6). The analysis of *vegfc* RNA transcript levels via in situ hybridization showed similar results in some domains of *vegfc* expression (Fig. 4b–d). *mt2* morphants show reduced *vegfc* staining, especially in the region, where the PHBCs develop (Fig. 4c, arrowheads). Interestingly, the increase in *vegfc* RNA expression in *mt2*-injected embryos was also confined to specific domains, including the region of PHBC development (Fig. 4b–d, arrowheads), but not ubiquitously distributed (Fig. 4d). Therefore, our results show that Mt2 is required for regulating *vegfc*, e.g., during PHBC formation, but it is not sufficient to induce general *vegfc* expression ectopically. This can be further substantiated when comparing the WT expression patterns of *vegfc* and *mt2*, showing that some domains of *vegfc* expression are in the same region as *mt2* expression, while there are also *vegfc* expression domains in areas not expressing high amounts of *mt2* (Fig. S6). We claim that the regulation of *vegfc* via Mt2 is specifically confined to specific vascular niches, such as the region of PHBC formation.

### Other metallothioneins cannot regulate *vegfc* expression

**Fig. 5:**
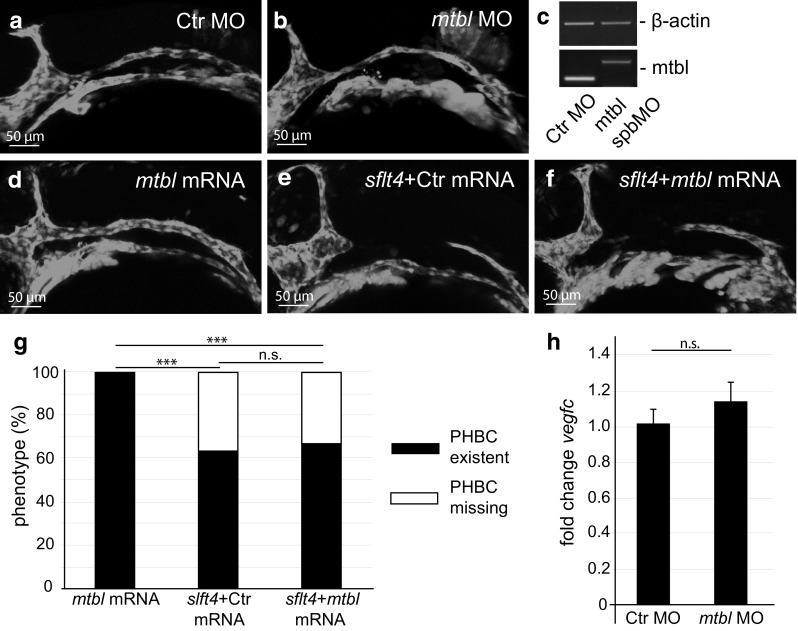
The *mt2* paralogue *metallothionein*-*B*-*like* (*mtbl*) does not regulate PHBC formation. *mtbl* MO-mediated deficiency does not affect PHBC formation. *Tg*(*kdrl:EGFP*)^*s843*^ embryos showed normal PHBC development at 24 hpf after injection of Ctr MO (**a**) or *mtbl* translation blocking MO (**b**). **c** Analysis of *mtbl* splicing efficiency in embryos injected with Ctr MO or *mtbl* spbMO. RT-PCR analysis showed a 327 bp amplicon in embryos injected with *mtbl* spbMO, while functional splicing led to a 169 bp amplicon in Ctr MO-injected embryos. *mtbl* overexpression failed to rescue PHBC formation defects induced by overexpression of a Vegfc ligand trap (*sflt4* overexpression). Overexpression of *mtbl* via mRNA injection (**d**) resulted in normal development of the PHBC, while *sflt4* mRNA injection resulted in PHBC formation failure (**e**). This phenotype could not be rescued through co-injection of *mtbl* mRNA (**f**). Quantification comparing the percentages of embryos lacking the PHBC, with *n* = 350, ***P < 0.001; n.s., not significant (**g**). The analysis was done with *Tg*(*kdrl:EGFP*)^*s843*^ embryos. **h** qPCR analysis of *mtbl* morphants showed no significant increase in the *vegfc* transcript. *Error bars* show SEM; n.s., not significant

To get more mechanistic insight how Mt2 could regulate *vegfc* expression, we questioned whether *vegfc* expression regulation could be a consequence of a cellular stress and hence would require the detoxifying features characteristic to all Metallothioneins (Mts). Therefore, we performed knockdown and overexpression experiments using another Metallothionein family member, *metallothionein*-*B*-*like* (*mtbl*; Fig. 5). To analyze *mtbl*-deficient embryos, we used again both translation and splice blocking MOs for our analysis and validated the functionality of the spbMO using RT-PCR (Fig. 5c). Even though *mtbl* deficiency led to defective development of the CCVs and Ses (Fig. S7), *mtbl* morphants showed normal PHBC development (Fig. 5b), indicating that during normal embryonic development Mt2 is specifically required for regulating *vegfc* expression. We next analyzed whether, as shown for Mt2, excess amounts of ectopic Mtbl could compensate for Vegfc ligand depletion by the ligand trap *sflt4*. While injection of *sflt4* mRNA again provoked defective PHBC development (Fig. 5e), co-injection with *mtbl* mRNA did not rescue this phenotype (Fig. 5f, g). Furthermore, *vegfc* transcripts were not significantly changed upon knockdown of *mtbl* (Fig. 5h). These results indicate that the regulation of *vegfc* transcription by Mt2 is not based on its Metallothionein characteristics and therefore not part of a cellular stress response, but rather represents an additional specific function of Mt2.

## Discussion

In this study, we showed that Mt2 regulates developmental angiogenesis in zebrafish by regulating *vegfc* mRNA expression. Vegfc regulates EC migration as a chemoattractant, e.g., by guiding ECs in the PHBCs [9, 11], and indeed, we show that correct migration of the PHBCs was perturbed by deletion of *mt2*. Additionally, Vegfc regulates EC proliferation [13], which was also perturbed in *mt2*-deficient zebrafish embryos.

We analyzed the role of Mt2 in zebrafish angiogenesis using MO-mediated Mt2 ablation as well as by using TALENs to introduce mutations in the zebrafish *mt2* gene. While we observed the same phenotypes in morphants as well as mutants, the phenotypes occurred at different frequencies between morphants and even between different hypothetical null mutants of *mt2* (see Table 1). Multiple mechanisms have been discussed to explain differences between mutant and morphant phenotypes: reinitiation at a downstream AUG or at an alternative start codon, exon skipping or the upregulation of other compensatory genes [63]. From the *mt2* sequence we can exclude reinitiation at a downstream AUG or exon skipping as potential mechanisms. We cannot predict whether there would be reinitiation at non-AUG start codons. However, we here provided a detailed analysis demonstrating that differences in mRNA stability, caused by NMD-mediated decay of the transcript, might account for the variability of the observed phenotypes. Our experiments show that a stronger efficiency of NMD led to a weaker penetrance of the phenotype, which might indicate transcript-level-based regulation of compensatory mechanisms in the embryo.

Additionally, even *vegfc* null mutants do not show full penetrance in failing to form the PHBC (Table 1, supplementary material [11]); therefore, embryos with a reduction in *vegfc* expression through *mt2* deficiency are not likely to display higher phenotypic frequencies.

In our study we identified a role for Mt2 in regulating angiogenesis upstream of transcriptional regulation of *vegfc* expression.

While in the zebrafish only two *mt* genes exist, in mammals there are at least four different gene families with differentially expressed isoforms [64]. Analysis of the amino acid sequence via UniProt revealed highest identity of the zebrafish Mt2 to the human and mouse MT1, closely followed by the human and mouse MT2. Mammalian *Mt1* and *Mt2* are supposedly very similar in their function [26] and have previously been implicated to be involved in angiogenic processes. The MZ*mt2* zebrafish knockout led to impaired development of major vessels, such as the PHBCs, the CCVs and the Ses and MZ*mt2*-deficient embryos died during larval stages. The murine *Mt1/2* double knockout in contrast was viable [36] and only displayed angiogenesis defects when challenged, e.g., by cortical freeze injury or femoral artery ligation [38–40]. As in zebrafish maternal message was capable of compensating *mt2* deficiency during embryonic angiogenesis, most likely in mammals other MT family might be able to compensate *Mt1/2* deficiency during development. However, a link to angiogenesis has also been established for human Mts in vitro [65].

We demonstrated that Mt2 but not Mtbl regulates angiogenesis upstream of *vegfc* transcription. Mt family members are involved in regulating a large number of developmental processes, including cell survival, cell proliferation, migration, scavenging of reactive oxygen species, and modulating the immune response. Most of these capabilities have been attributed to the metal-binding capabilities, resulting, e.g., in removal of cofactor ions such as zinc [26, 30, 66]. The zebrafish Mtbl is capable of fulfilling these MT family member functions, but does not rescue PHBC development in *mt2* morphants or *vegfc* ligand reduced embryos. We present here the first evidence for an additional role of zebrafish Mt2 in regulating *vegfc* expression independent of Mt function. Interestingly, upregulation of different human MT isoforms was observed comparing the responses to physiological or hypoxic conditions [65]. This could be taken as an indication for differential regulatory functions of some human MT family proteins, independent of the functions common to all MTs.

We analyzed whether other transcript levels were regulated by zebrafish Mt2 in addition to *vegfc*. Neither the Vegfc regulator *ccbe1* expression, nor the Pdgf/Vegf family member *c*-*fos*-*induced growth factor* (*figf*) expression was altered. In contrast, *vegfa* expression seemed also regulated downstream of MT2 (data not shown). Reduced *Vegfa* RNA [40] and VEGFA protein levels [38] were reported in *Mt1/2*-deficient mice. While changes in Vegfc expression explained the PHBC and CCV phenotypes, reduction in Vegfa expression would account for the failures in Se formation, as deficiency in either Vegfa or its receptor Kdrl result in severe Se phenotypes [9, 67].

In sum, we have identified a novel role of MT2 in regulating angiogenesis by regulating *vegfc* transcription, which might be conserved in mammals.

We for the first time show that this regulatory role is specific to zebrafish Mt2 and represents a novel, non-canonical function of MT2, most likely not attributed to metal-binding capabilities of MT proteins.

## Electronic supplementary material

Supplementary Fig. S1 Angiogenesis of the CCVs and the Ses is impaired after MO mediated *mt2* ablation
(**a**–**c**) Confocal micrographs of the PHBC of Ctr MO (a) and *mt2* MO (b) or *mt2*spbMO (c) injected embryos. (**d**) Schematic illustration of the vasculature of a 32 hpf old zebrafish embryo with annotation of the region of the CCV and the Ses. (**e**) Schematic close-up of the CCV and the adjacent ACV and PCV indicates the areas, which were calculated for the analysis. (**f**–**h**) Confocal micrographs of the CCV of Ctr MO (f) and *mt2* MO (g) or *mt2* spbMO (h) injected embryos show defective CCV development for *mt2* deficient zebrafish embryos. (**i**) Total EC numbers of the ACV/PCV compared to the CCV were normalized for both Ctr and *mt2* MO or *mt2* spbMO and show reduced cell numbers especially for the CCV of *mt2* morphants. n = 30, ***P < 0.001, **P < 0.01; error bars indicate s.e.m. (**j**) Quantification of ECs in the ACV, PCV and CCV shows a reduced overall EC number for all vessel areas. (**k**–**p**) Ses of *mt2* MO or *mt2* spbMO injected embryos at 24 and 48 hpf (l, m, o, p) are missing or malformed compared to the Ses of Ctr Mo injected embryos (k, n). (**q**) Quantification of ECs in the Ses shows reduced cell numbers for *mt2* deficient zebrafish embryos. The average of ECs of the Ses in somites nine to 14 above the yolk extension was calculated. The analyses were performed with *Tg(kdrl:EGFP)* ^*s843*^ zebrafish embryos. n = 50, ***P < 0.001; error bars indicate s.e.m.; Se: intersegmental vessels; ACV: anterior cardinal vein; PCV: posterior cardinal vein; CCV: common cardinal vein. Supplementary material 1 (PDF 3651 kb)

Supplementary Fig. S2 The proliferation of ECs is reduced in *mt2* morphant embryos
(**a**,**b**) BrdU incorporation from 24–32 hpf leads to fewer cells in *mt2* morphants (b) and vegfchu6410 mutant embryos (a) compared to WT (A) in the CCV at 32 hpf. BrdU positive cells are labeled in red, ECs are visualized by GFP expression in *Tg(kdrl:EGFP)* ^*s843*^ in green. Single channels for BrdU (a’,b’) and GFP (a’’,b’’) reveal double positive cells. White arrowheads visualize these events in representative picture. A white line marks the border of the CCVs. (**c**) Quantification of BrdU positive cells in the CCV shows significantly less proliferation in *mt2* morphants embryos compared to WT. Black bars represent the percentage of BrdU positive cells in relation to total EC numbers in the CCVs of *mt2* morphants and WT zebrafish embryos. n = 13, ***P < 0.001; error bars indicate s.e.m. Supplementary material 2 (PDF 8258 kb)

Supplementary Fig. S3 TALEN mediated genomic changes in the different *mt2* mutants.
(**a**) WT sequence of *mt2* with annotation of the individual exons. The start codon is marked by a red line. The dashed black box indicates the sequence region targeted and shown in Figure S3b. (**b**) TALENs were used to induce three different mutations in exon 1 of *mt2*. The *mt2* ^*mu289*^ has a 6 bp deletion. The *mt2* ^*mu290*^ sequence has a 2 bp point mutation as well as an 8 bp insertion. The *mt2* ^*mu292*^ sequence has a 15 bp deletion including deletion of the start codon. Both mutations in *mt2* ^*mu290*^ and *mt2* ^*mu292*^ cause frameshifts leading to nonsense proteins. Supplementary material 3 (PDF 129 kb)

Supplementary Fig. S4 Angiogenesis of the CCVs and the Ses is impaired in *mt2* mutantembryos
(**a**–**e**) The CCVs of *mt2* mutant embryos display significantly fewer ECs at 32 hpf. MZ*mt2* ^*mu289*^ and MZ*mt2* ^*mu290*^ were analyzed by visualizing GFP expression from *Tg(fli1a:EGFP)* ^*y1*^; MZ*mt2* ^*mu292*^ mutant embryos were analyzed by visualizing GFP expression from *Tg(kdrl:EGFP)* ^*s843*^. n = 56, ***P < 0.001; the graph shows mean with error bars indicating s.e.m. (**f**–**i**) Defects in the Ses at 24 hpf were observed in MZ*mt2* ^*mu290*^ (**h**) and MZ*mt2* ^*mu292*^ (**i**) mutant embryos, but not in WT (**f**) or MZ*mt2* ^*mu289*^ mutant (**g**) embryos. Mz*mt2* ^*mu290*^ and MZ*mt2* ^*mu292*^ mutant Ses were stalled at the level of the horizontal myoseptum. Supplementary material 4 (PDF 2057 kb)

Supplementary Fig. S5 Blocking of NMD aggravates the phenotype in MZ*mt2* mutants
(**a**–**e**) WT (a,c) and MZ*mt2* ^*mu290*^ mutant zebrafish (b,d) were injected with NMD blocking MOs (c,d). While no effect could be observed for WT zebrafish embryos (c), significantly more MZ*mt2* ^*mu290*^ mutant zebrafish embryos displayed PHBC defects upon NMD blockage (d). The analysis was performed visualizing vascular specific GFP expression from *Tg(fli1a:nEGFP)* ^*y7*^. Black bars label percent of embryos with complete PHBCs, white bars label percent of embryos lacking the PHBC, statistical significance was calculated with the Chi Square test, n = 622, ***P < 0.001; n.s., not significant. (**f**) Models relating phenotype and NMD in the different *mt2* mutants. Normal translation is occurring in WT and MZ*mt2* ^*mu289*^ mutant zebrafish embryos. A stop in proximity to the polyA tail and the release factor (RF) allow the completion of the translation process. Premature stop codons lead to stalling of the ribosomes in both MZ*mt2* ^*mu290*^ and MZ*mt2* ^*mu292*^ zebrafish embryos. The NMD machinery is recruited and induces degradation of the mRNA. Differential amounts of NMD might result in alterations in compensation mechanisms. The curved black line illustrates the mRNA. The ribosome is shown in yellow. Stop codons are marked with a red box, premature stop codons are further marked with red asterisks. Proteins of the NMD machinery are shown in green and blue boxes. Upf: Up-frameshift, Smg: suppressor with morphological effect on genitalia, RF: Releasefactor. The red asterisk used for MZ*mt2* ^*mu289*^ mutants indicates the 2AA deletion. Supplementary material 5 (PDF 1306 kb)

Supplementary Fig. S6 Transcript expression analysis in embryos *mt2* deficient or overexpressing embryos.
(**a**) qPCR analysis of *fli1a* and *myod1* transcript levels in *mt2* morphants, Mz*mt2* ^*mu290*^ mutants and *mt2* mRNA injected embryos compared to Ctr embryos. In contrast to *vegfc* transcripts, which were significantly regulated in correlation with *mt2* transcript levels (Fig 4a), *fli1a* and *myod1* transcripts were not significantly changed. Only the *myod1* transcript shows a minimal upregulation in the *mt2* morphants. (**b**) qPCR analysis of *mt2* injected *vegfc* ^*hu6410-/-*^ mutant zebrafish shows transcript changes of *vegfc* similar to those in *mt2* injected *Tg(kdrl:EGFP)* ^*s843*^ (Fig 4a). Supplementary material 6 (PDF 120 kb)

Supplementary Fig. S7 Transcript expression patterns are similar for *mt2* and *vegfc*.
(*a*–**j**) RNA Expression patterns analyzed by in situ hybridization for *mt2* (a-e) and *vegfc* (f-j) from 18 to 32 hpf in WT zebrafish embryos. Lateral views focusing on anterior expression (including the area of the PHBC). Supplementary material 7 (PDF 6471 kb)

Supplementary Fig. S8 Angiogenesis of the CCVs and the Ses is partially impaired in *mtbl* morphant embryos.
(**a**,**b**) The CCVs of *mtbl* morphant embryos (b) were thinner compared to Ctr MO injected zebrafish embryos (a) at 32 hpf. (**c**–**f**) Mild defects in the Ses of *mtbl* morphants were observable at 24 hpf (d) and 32 hpf (f), while Ctr morphants (c,e) develop normally. The Ses of *mtbl* morphants are stalled at the horizontal myoseptum and the trunk shows mild bending of the body axis. All embryos were analyzed by visualizing GFP expression from *Tg(kdrl:EGFP)* ^*s843*^. Supplementary material 8 (PDF 1778 kb)

Supplementary Movie S1 Ctr morphants show normal development of the PHBC
The proper development of the PHBC in Ctr MO injected *Tg(kdrl:EGFP)* ^*s843*^ zebrafish embryos is visualized from 17 to 30 hpf. The LDA has already formed when ECs of the PHBCs start to migrate from anterior and posterior clusters to connect at approximately 23 hpf. There are no extensive migration movements of ECs of the PHBC between 23 and 30 hpf. A confocal stack was taken every 15 minutes. Supplementary material 9 (MPG 1922 kb)

Supplementary Movie S2 *mt2* morphants do not develop a functional PHBC
The development of the PHBC in *mt2* MO injected *Tg(kdrl:EGFP)* ^*s843*^ zebrafish embryos between 17 and 30 hpf is severely impaired. The LDA forms normally similar to Ctr zebrafish embryos. The PHBC however does not connect until 30 hpf. The ECs are motile and send filopodia, but the directed migration of the ECs is restricted. Furthermore the anterior cluster, where the PHBC, the PMBC, the MceV and the OV connect is thickened and malformed. The posterior cluster shows more ECs as well. Since EC numbers are not altered and no ECs undergoing apoptosis can be observed, the impaired migration of ECs is causing the phenotype. A confocal stack was taken every 15 minutes. Supplementary material 10 (MPG 1794 kb)
