# Supplementary figures and images for: Metallothionein 2 regulates endothelial cell migration through transcriptional regulation of vegfc expression
Source: Angiogenesis. 2015 Jul 22;18(4):463–75. doi: 10.1007/s10456-015-9473-6 (PMC4596909; doi:10.1007/s10456-015-9473-6)

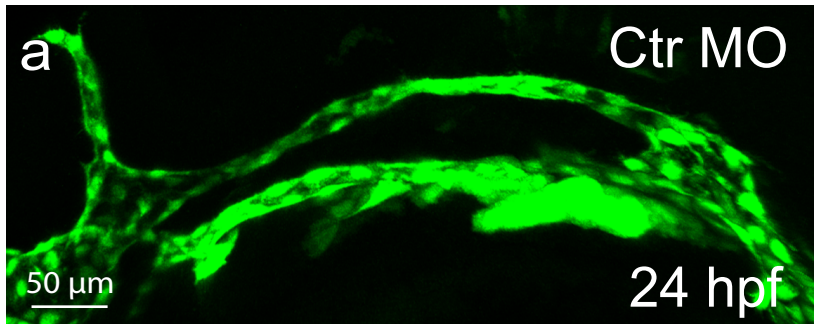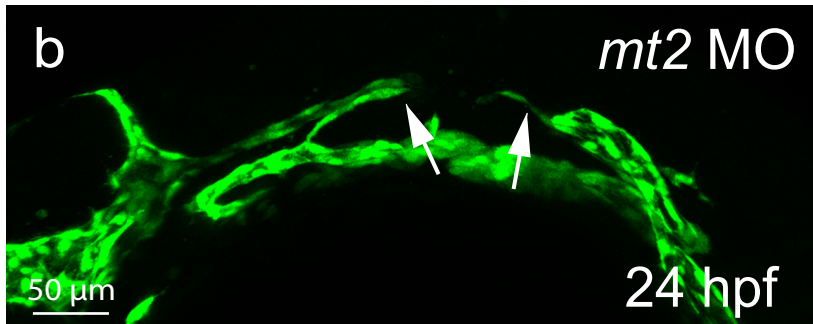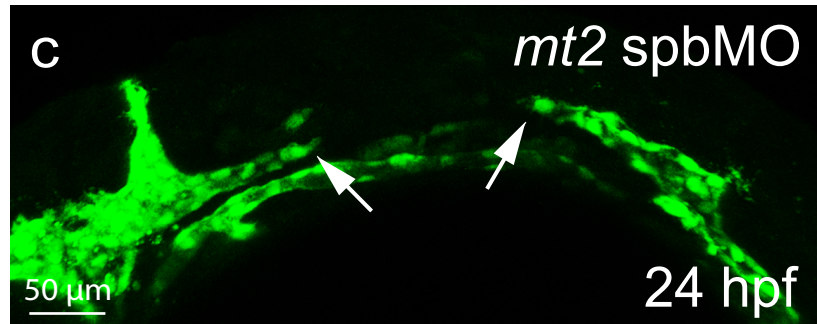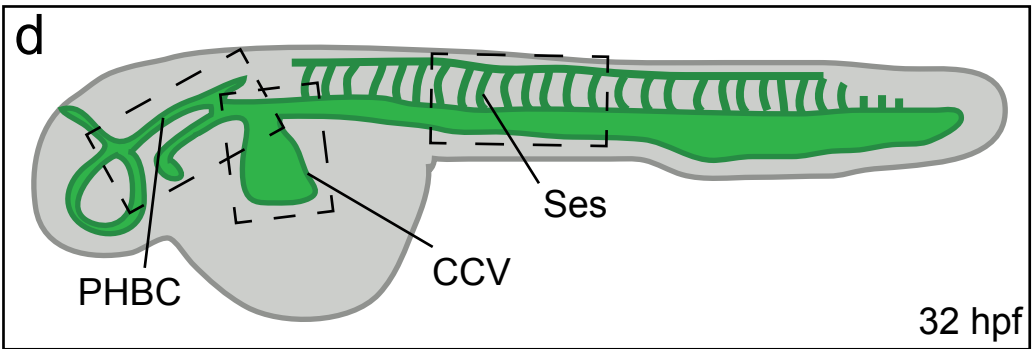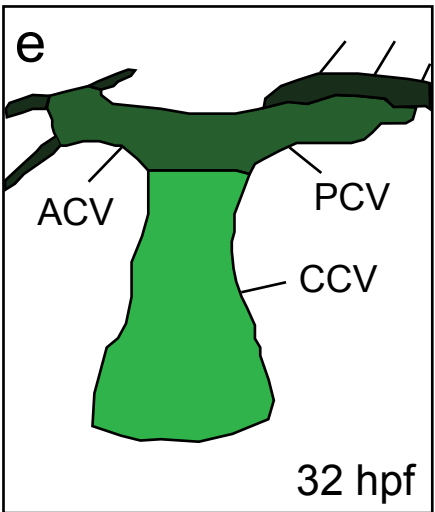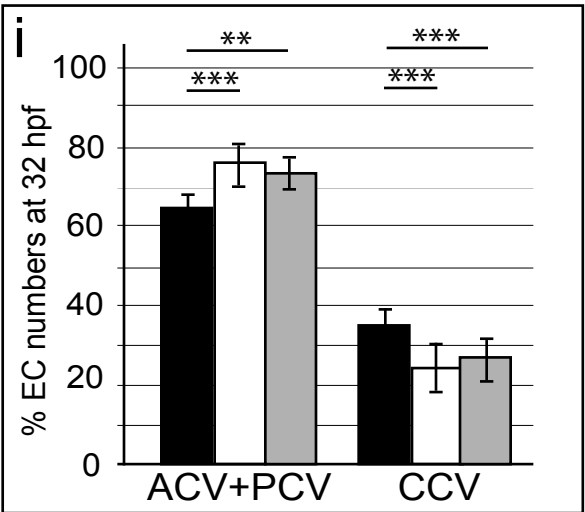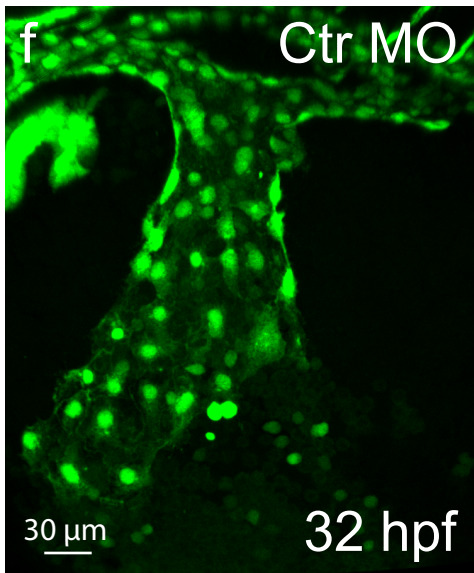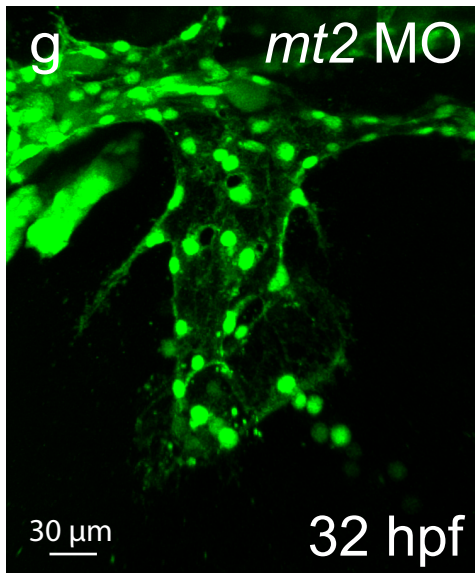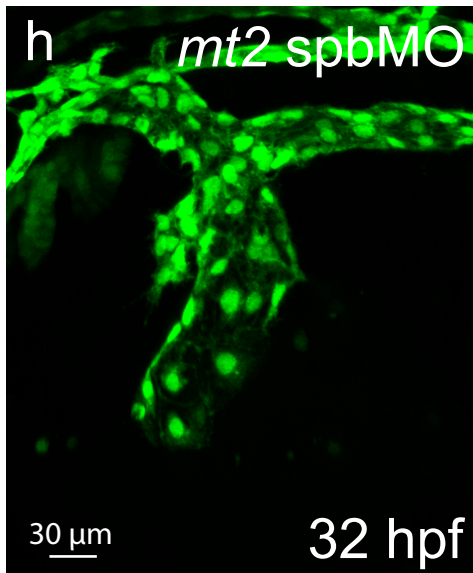

**j**

|                 | ECs Ctr MO | ECs <i>mt2</i> MO | ECs <i>mt2</i> spbMO |
|-----------------|------------|-------------------|----------------------|
| ACV + PCV       | 58         | 51                | 48                   |
| CCV             | 32         | 16                | 18                   |
| ACV + PCV + CCV | 90         | 67                | 66                   |

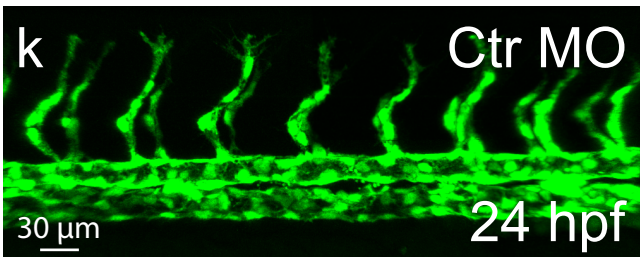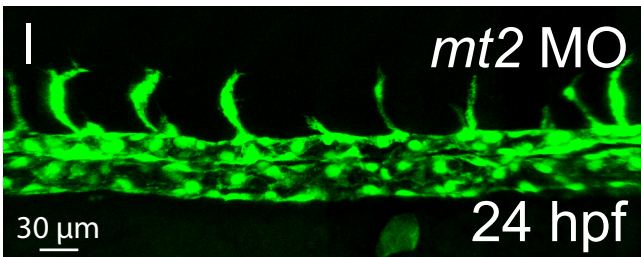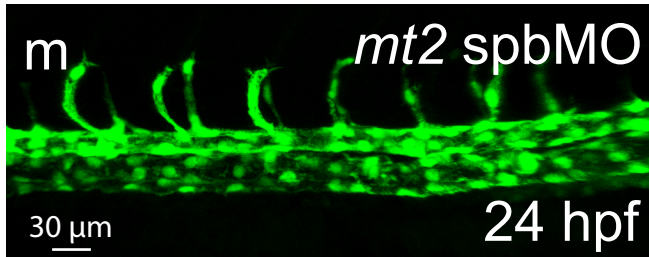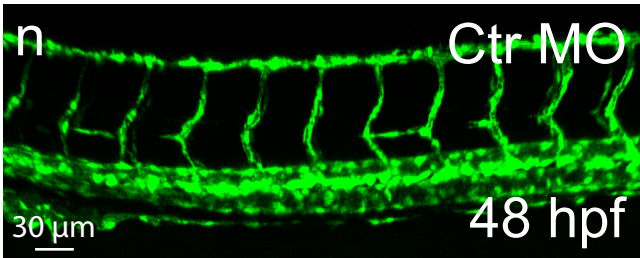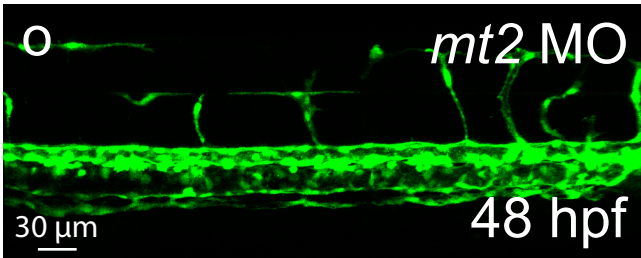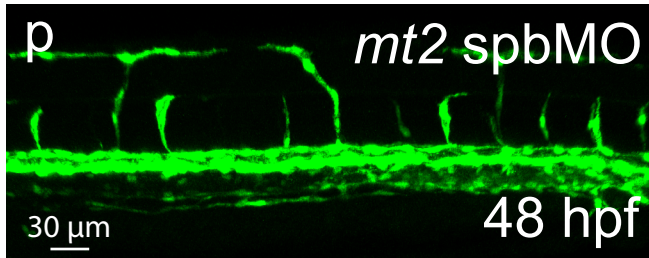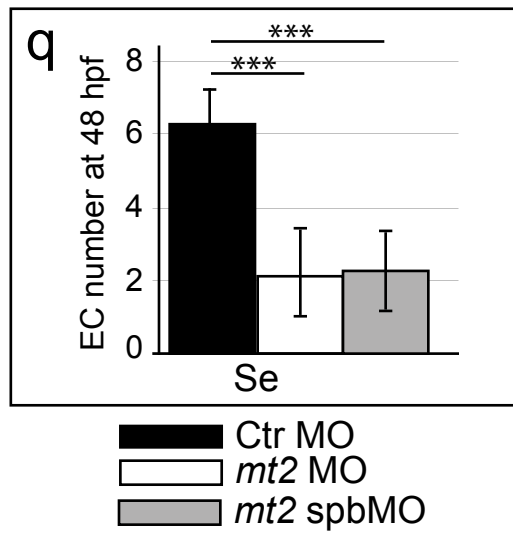

Supplement: Supplementary file 1 — Angiogenesis of the CCVs and the Ses is impaired after MO mediated mt2 ablation (a–c) Confocal micrographs of the PHBC of Ctr MO (a) and mt2 MO (b) or mt2spbMO (c) injected embryos. (d) Schematic illustration of the vasculature of a 32 hpf old zebrafish embryo with annotation of the region of the CCV and the Ses. (e) Schematic close-up of the CCV and the adjacent ACV and PCV indicates the areas, which were calculated for the analysis. (f–h) Confocal micrographs of the CCV of Ctr MO (f) and mt2 MO (g) or mt2 spbMO (h) injected embryos show defective CCV development for mt2 deficient zebrafish embryos. (i) Total EC numbers of the ACV/PCV compared to the CCV were normalized for both Ctr and mt2 MO or mt2 spbMO and show reduced cell numbers especially for the CCV of mt2 morphants. n = 30, ***P < 0.001, **P < 0.01; error bars indicate s.e.m. (j) Quantification of ECs in the ACV, PCV and CCV shows a reduced overall EC number for all vessel areas. (k–p) Ses of mt2 MO or mt2 spbMO injected embryos at 24 and 48 hpf (l, m, o, p) are missing or malformed compared to the Ses of Ctr Mo injected embryos (k, n). (q) Quantification of ECs in the Ses shows reduced cell numbers for mt2 deficient zebrafish embryos. The average of ECs of the Ses in somites nine to 14 above the yolk extension was calculated. The analyses were performed with Tg(kdrl:EGFP) s843 zebrafish embryos. n = 50, ***P < 0.001; error bars indicate s.e.m.; Se: intersegmental vessels; ACV: anterior cardinal vein; PCV: posterior cardinal vein; CCV: common cardinal vein. Supplementary material 1 (PDF 3651 kb) [file 10456_2015_9473_MOESM1_ESM.pdf]

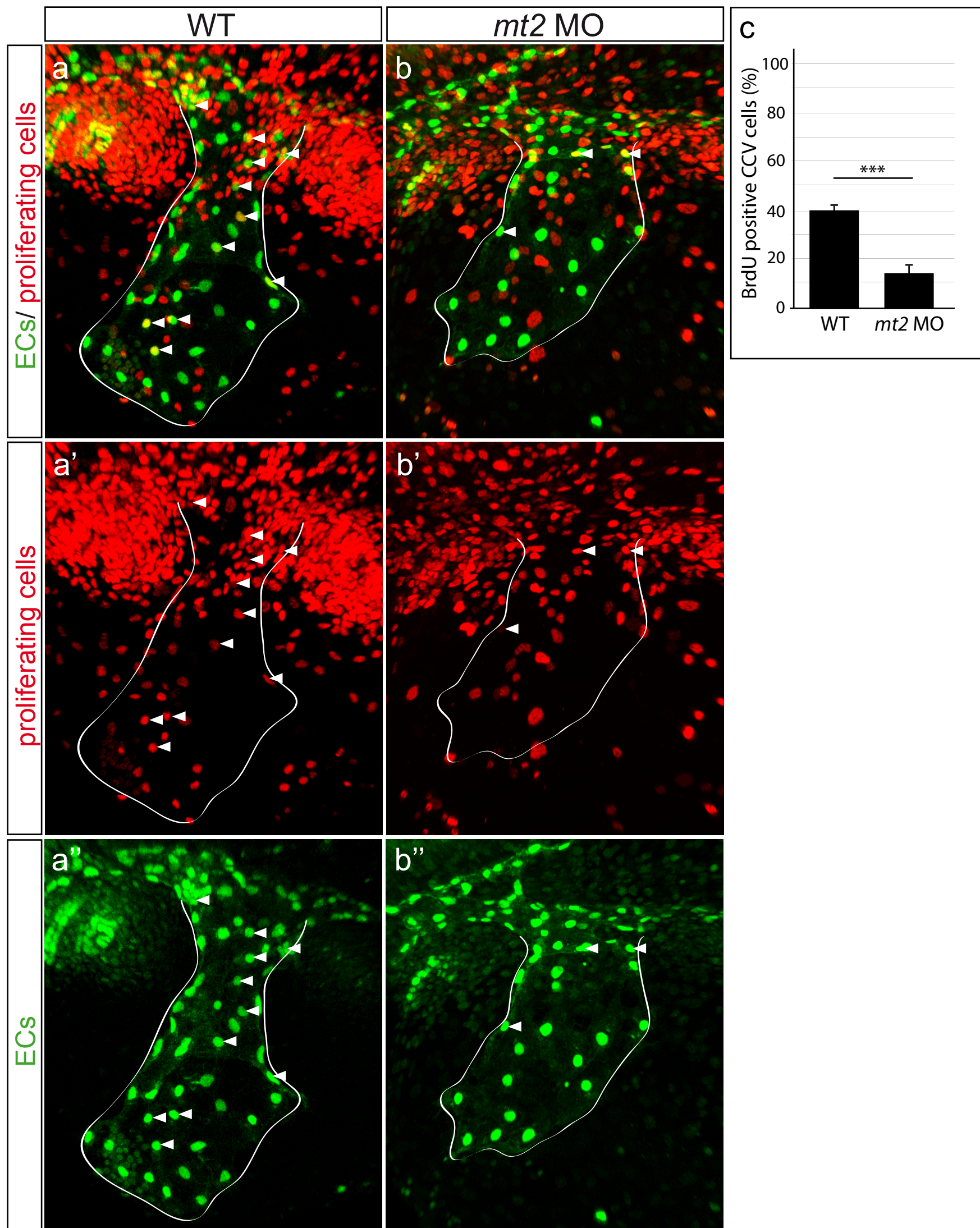

Supplement: Supplementary file 2 — The proliferation of ECs is reduced in mt2 morphant embryos (a,b) BrdU incorporation from 24–32 hpf leads to fewer cells in mt2 morphants (b) and vegfchu6410 mutant embryos (a) compared to WT (A) in the CCV at 32 hpf. BrdU positive cells are labeled in red, ECs are visualized by GFP expression in Tg(kdrl:EGFP) s843 in green. Single channels for BrdU (a’,b’) and GFP (a’’,b’’) reveal double positive cells. White arrowheads visualize these events in representative picture. A white line marks the border of the CCVs. (c) Quantification of BrdU positive cells in the CCV shows significantly less proliferation in mt2 morphants embryos compared to WT. Black bars represent the percentage of BrdU positive cells in relation to total EC numbers in the CCVs of mt2 morphants and WT zebrafish embryos. n = 13, ***P < 0.001; error bars indicate s.e.m. Supplementary material 2 (PDF 8258 kb) [file 10456_2015_9473_MOESM2_ESM.pdf]

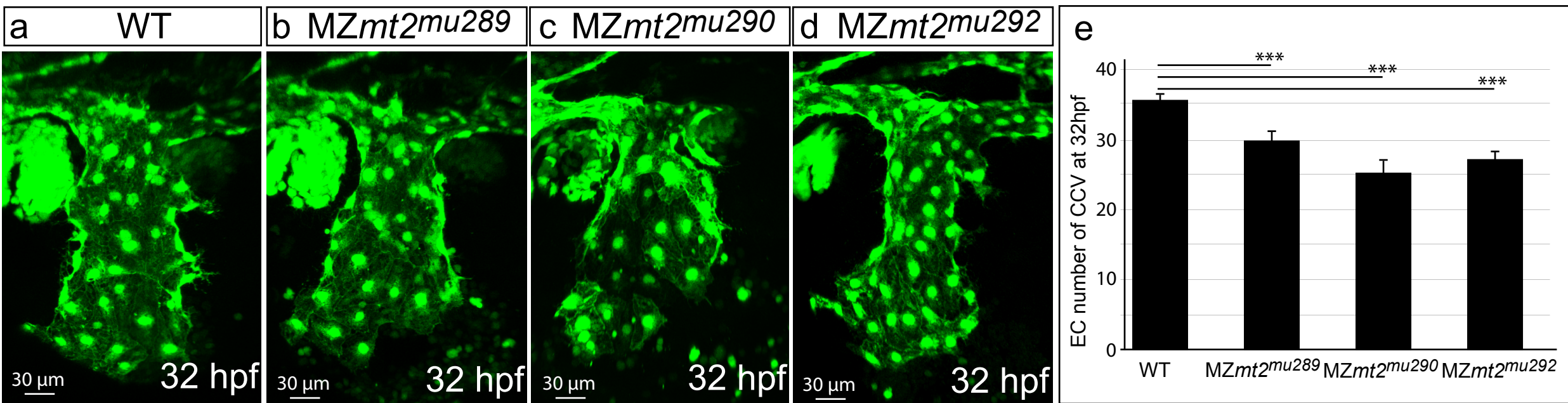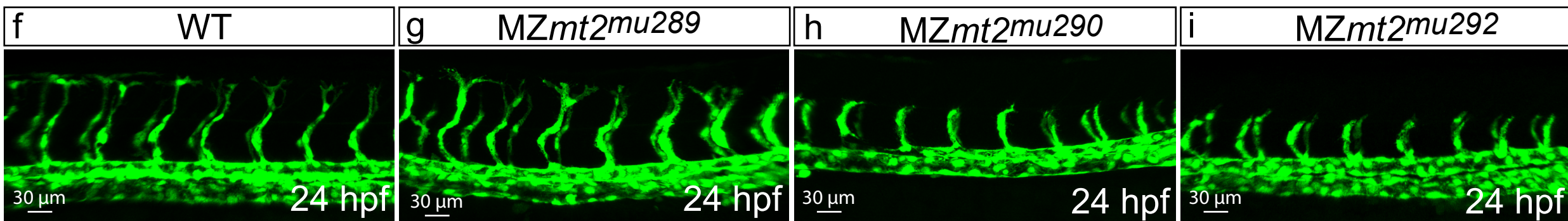

Supplement: Supplementary file 4 — Angiogenesis of the CCVs and the Ses is impaired in mt2 mutantembryos (a–e) The CCVs of mt2 mutant embryos display significantly fewer ECs at 32 hpf. MZmt2 mu289 and MZmt2 mu290 were analyzed by visualizing GFP expression from Tg(fli1a:EGFP) y1; MZmt2 mu292 mutant embryos were analyzed by visualizing GFP expression from Tg(kdrl:EGFP) s843. n = 56, ***P < 0.001; the graph shows mean with error bars indicating s.e.m. (f–i) Defects in the Ses at 24 hpf were observed in MZmt2 mu290 (h) and MZmt2 mu292 (i) mutant embryos, but not in WT (f) or MZmt2 mu289 mutant (g) embryos. Mzmt2 mu290 and MZmt2 mu292 mutant Ses were stalled at the level of the horizontal myoseptum. Supplementary material 4 (PDF 2057 kb) [file 10456_2015_9473_MOESM4_ESM.pdf]

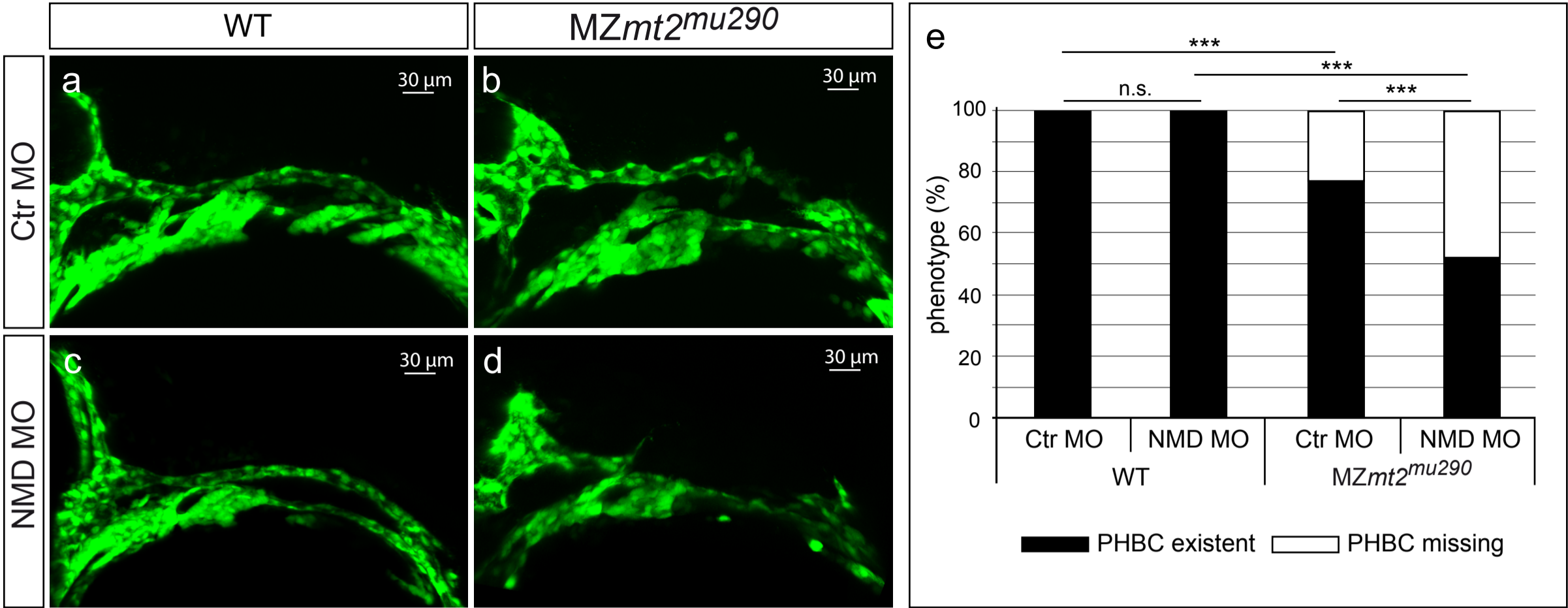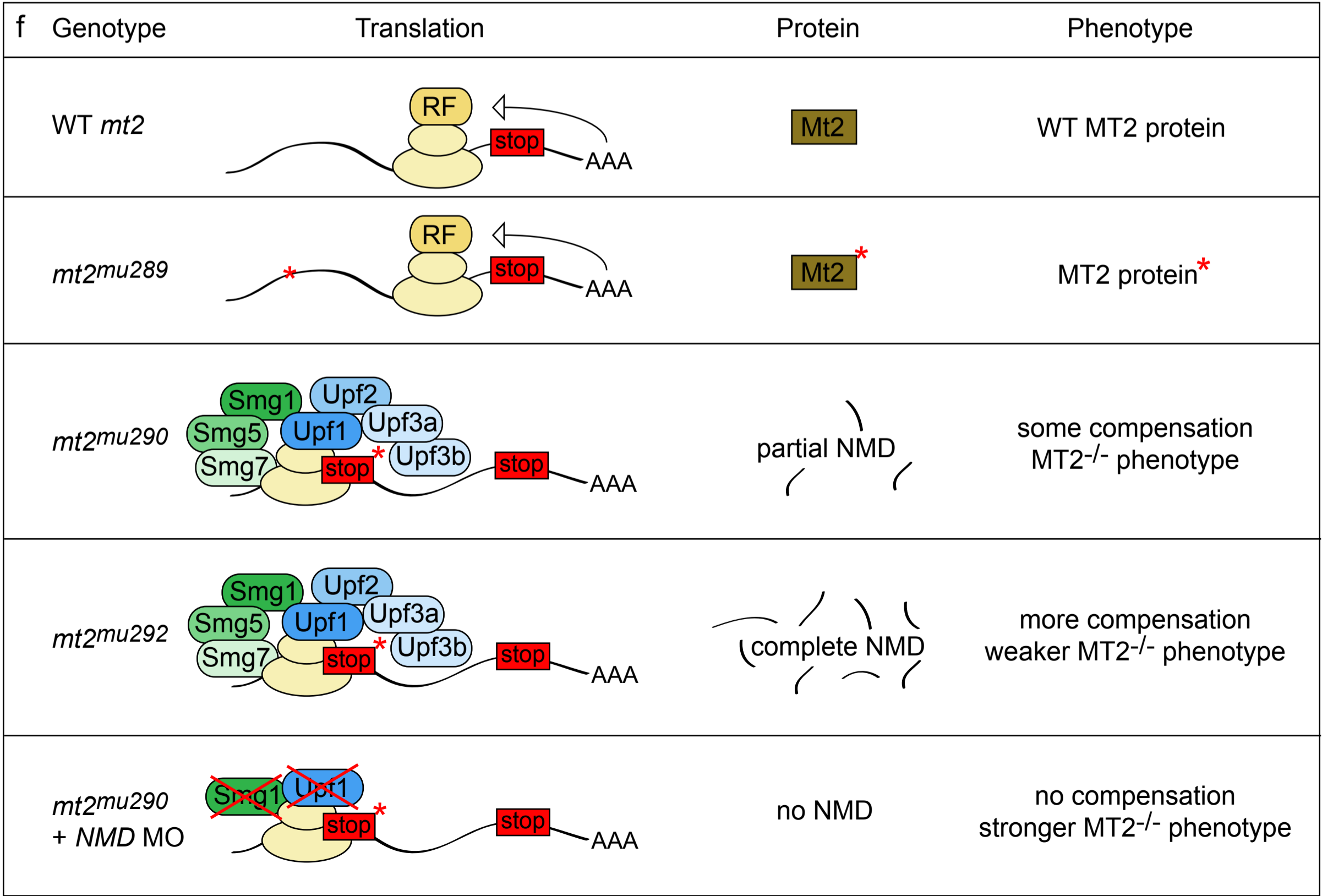

Supplement: Supplementary file 5 — Blocking of NMD aggravates the phenotype in MZmt2 mutants (a–e) WT (a,c) and MZmt2 mu290 mutant zebrafish (b,d) were injected with NMD blocking MOs (c,d). While no effect could be observed for WT zebrafish embryos (c), significantly more MZmt2 mu290 mutant zebrafish embryos displayed PHBC defects upon NMD blockage (d). The analysis was performed visualizing vascular specific GFP expression from Tg(fli1a:nEGFP) y7. Black bars label percent of embryos with complete PHBCs, white bars label percent of embryos lacking the PHBC, statistical significance was calculated with the Chi Square test, n = 622, ***P < 0.001; n.s., not significant. (f) Models relating phenotype and NMD in the different mt2 mutants. Normal translation is occurring in WT and MZmt2 mu289 mutant zebrafish embryos. A stop in proximity to the polyA tail and the release factor (RF) allow the completion of the translation process. Premature stop codons lead to stalling of the ribosomes in both MZmt2 mu290 and MZmt2 mu292 zebrafish embryos. The NMD machinery is recruited and induces degradation of the mRNA. Differential amounts of NMD might result in alterations in compensation mechanisms. The curved black line illustrates the mRNA. The ribosome is shown in yellow. Stop codons are marked with a red box, premature stop codons are further marked with red asterisks. Proteins of the NMD machinery are shown in green and blue boxes. Upf: Up-frameshift, Smg: suppressor with morphological effect on genitalia, RF: Releasefactor. The red asterisk used for MZmt2 mu289 mutants indicates the 2AA deletion. Supplementary material 5 (PDF 1306 kb) [file 10456_2015_9473_MOESM5_ESM.pdf]

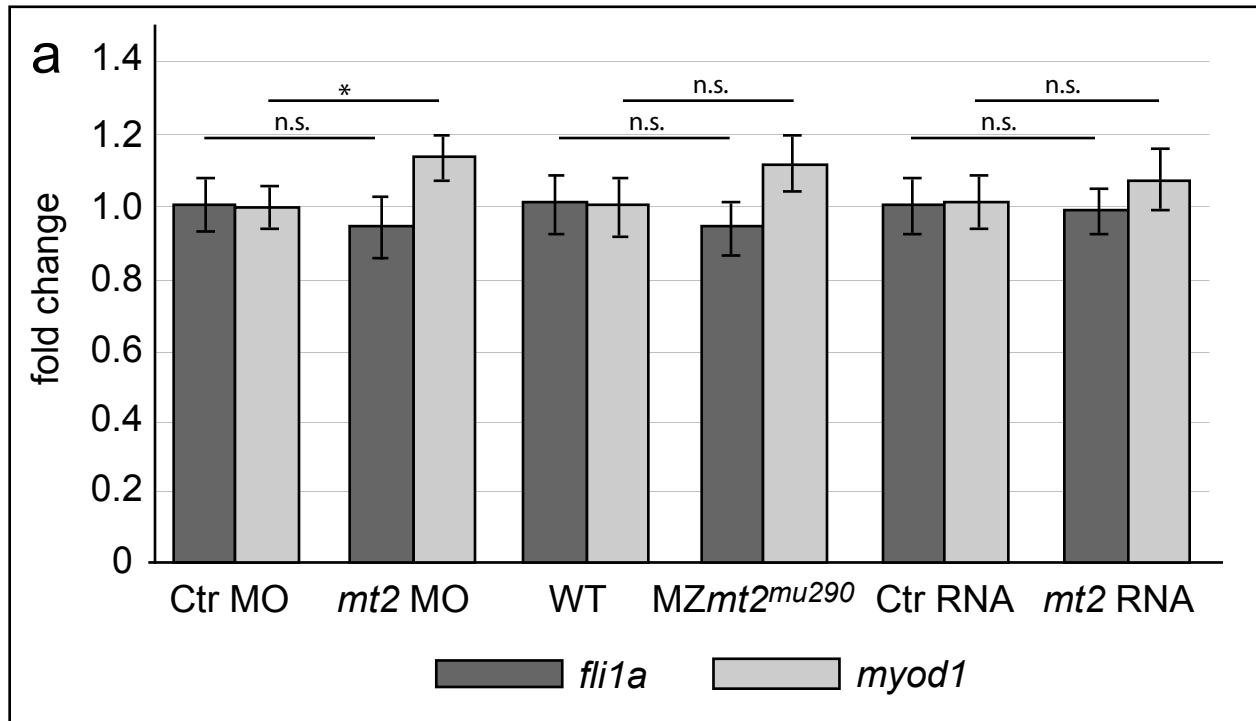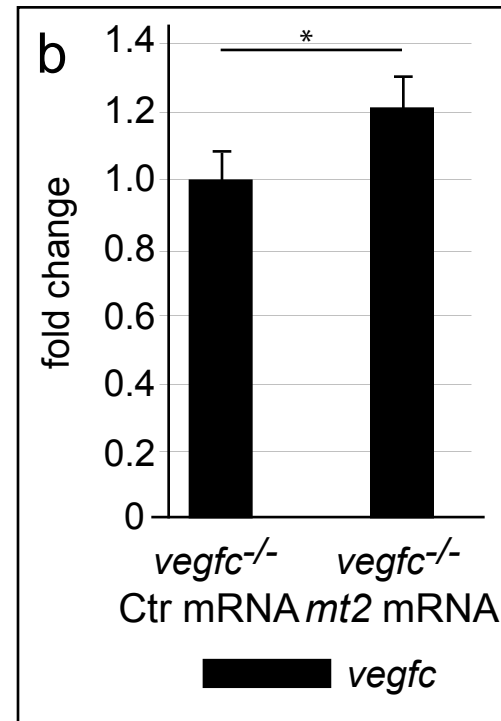

Supplement: Supplementary file 6 — Transcript expression analysis in embryos mt2 deficient or overexpressing embryos. (a) qPCR analysis of fli1a and myod1 transcript levels in mt2 morphants, Mzmt2 mu290 mutants and mt2 mRNA injected embryos compared to Ctr embryos. In contrast to vegfc transcripts, which were significantly regulated in correlation with mt2 transcript levels (Fig 4a), fli1a and myod1 transcripts were not significantly changed. Only the myod1 transcript shows a minimal upregulation in the mt2 morphants. (b) qPCR analysis of mt2 injected vegfc hu6410-/- mutant zebrafish shows transcript changes of vegfc similar to those in mt2 injected Tg(kdrl:EGFP) s843 (Fig 4a). Supplementary material 6 (PDF 120 kb) [file 10456_2015_9473_MOESM6_ESM.pdf]

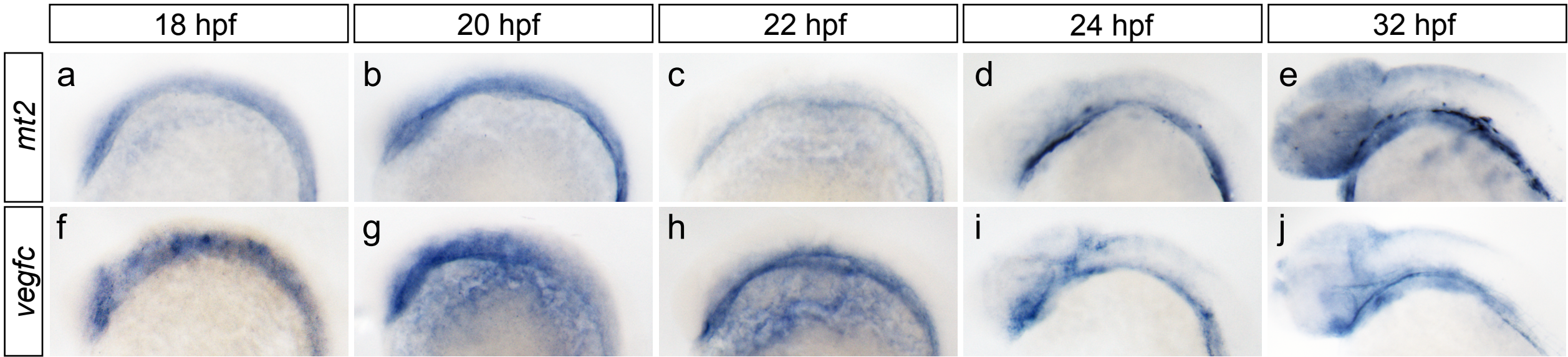

Supplement: Supplementary file 7 — Transcript expression patterns are similar for mt2 and vegfc. (a–j) RNA Expression patterns analyzed by in situ hybridization for mt2 (a-e) and vegfc (f-j) from 18 to 32 hpf in WT zebrafish embryos. Lateral views focusing on anterior expression (including the area of the PHBC). Supplementary material 7 (PDF 6471 kb) [file 10456_2015_9473_MOESM7_ESM.pdf]

Ctr MO

*mtbl* MO

Ctr MO

*mtbl* MO

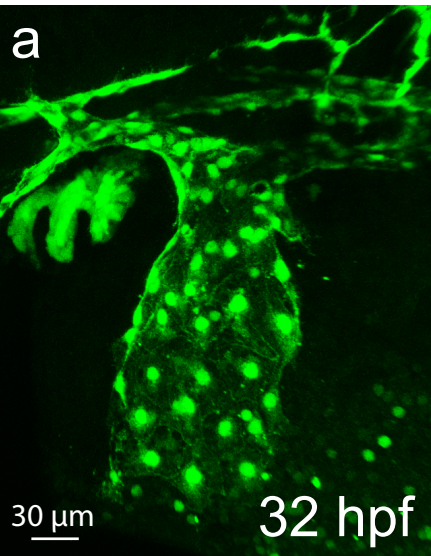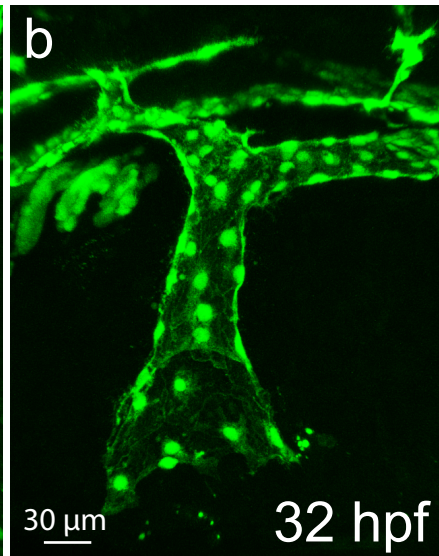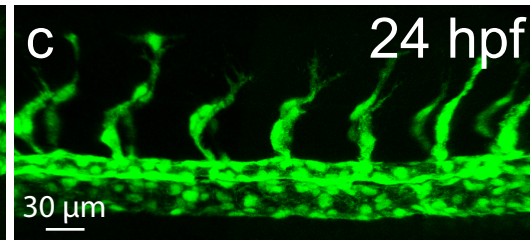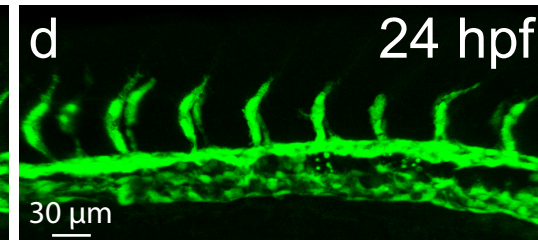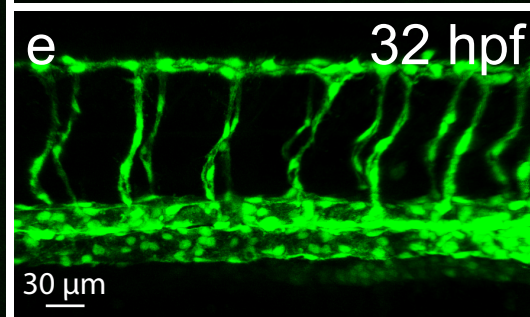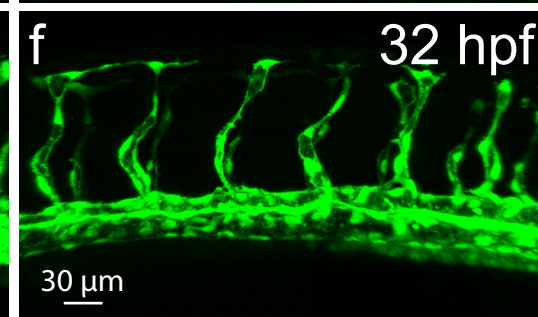

Supplement: Supplementary file 8 — Angiogenesis of the CCVs and the Ses is partially impaired in mtbl morphant embryos. (a,b) The CCVs of mtbl morphant embryos (b) were thinner compared to Ctr MO injected zebrafish embryos (a) at 32 hpf. (c–f) Mild defects in the Ses of mtbl morphants were observable at 24 hpf (d) and 32 hpf (f), while Ctr morphants (c,e) develop normally. The Ses of mtbl morphants are stalled at the horizontal myoseptum and the trunk shows mild bending of the body axis. All embryos were analyzed by visualizing GFP expression from Tg(kdrl:EGFP) s843. Supplementary material 8 (PDF 1778 kb) [file 10456_2015_9473_MOESM8_ESM.pdf]
