# Supplementary material for: Metallothionein 2 regulates endothelial cell migration through transcriptional regulation of vegfc expression
Source: Angiogenesis. 2015 Jul 22;18(4):463–75. doi: 10.1007/s10456-015-9473-6 (PMC4596909; doi:10.1007/s10456-015-9473-6)
Supplement: Supplementary file 3 — TALEN mediated genomic changes in the different mt2 mutants. (a) WT sequence of mt2 with annotation of the individual exons. The start codon is marked by a red line. The dashed black box indicates the sequence region targeted and shown in Figure S3b. (b) TALENs were used to induce three different mutations in exon 1 of mt2. The mt2 mu289 has a 6 bp deletion. The mt2 mu290 sequence has a 2 bp point mutation as well as an 8 bp insertion. The mt2 mu292 sequence has a 15 bp deletion including deletion of the start codon. Both mutations in mt2 mu290 and mt2 mu292 cause frameshifts leading to nonsense proteins. Supplementary material 3 (PDF 129 kb) [file 10456_2015_9473_MOESM3_ESM.pdf]

a DNA sequence WT *mt2*

GCCTCCAGCATCAACTCATTCACAAGCTGAGTGAACGATATTTCTAAGGAACTTTCAAGCTCTTTG
TGGATACTCTCTGGAAAAATGGACCCCTGCGAATGTGCCAAGACTGGAACTTGCAACTGTGGTGC
TACCTGCAAGTGCACTAATTGCCAGTGTACTACCTGCAAGAAGAGTTGTTGTTCTTGCTGCCCATC
TGGTTGCAGCAAGTGTGCCTCTGGCTGCGTCTGCAAAGGCAATTCCTGTGGCTCCAGCTGCTGT
CAATGAAGAGGTCAACGTGATGTTTTCTACAATGTGAATCTGTTTGTCTACTCCACGTCTGCGTTTT
GCATCGCATGATTGTCTTTTTTTTTTTATTTTTTATTACAAGATGATAAATGACCTCTTTGTTCTCAATC
TTGTCTGTTTAATGTTGCAAAAATCGTCTAACAAAGGCTAAAGAGGGAAAACGATGCACTGATTTGT
ACTGCTGCAAATAAGACTATCACGAGATGTCTTTAAAATGAAACTATTGTACGTTTTTCTGCCATGTC
CTTTGTCTAAAATAAATGGTGGTATTTATTTTTGTGTGGCTTGTTTTTGTAATTATTGGTA

Exon 1

Exon 2

Exon 3

area in b

start codon

b Mutations in *mt2* mutants

codon usage

DNA sequence WT *mt2*: AAAATG G A C CCCTGCGAATGTGCC
DNA sequence *mt2*^{mu289}: AAAATG - - - - - - TGTGCATGTGCC (6 bp deletion)
DNA sequence *mt2*^{mu290}: AATGTGCCAAGTCATCTCCCTGCGAATGTGCC (2 bp point mutation, 8 bp insertion)
DNA sequence *mt2*^{mu292}: A - - - - - - - - - - - - - - - - AATGTGCC (15 bp deletion)
